# Enhanced physical and cognitive performance in active duty Airmen: evidence from a randomized multimodal physical fitness and nutritional intervention

**DOI:** 10.1038/s41598-020-74140-7

**Published:** 2020-10-19

**Authors:** Christopher E. Zwilling, Adam Strang, Evan Anderson, Jennifer Jurcsisn, Erica Johnson, Tapas Das, Matthew J. Kuchan, Aron K. Barbey

**Affiliations:** 1 Decision Neuroscience Laboratory, University of Illinois, 405 North Mathews Avenue, Urbana, IL 61801 USA; 2 Beckman Institute for Advanced Science and Technology, University of Illinois, Urbana, IL USA; 3 Applied Neuroscience Branch, Wright Patterson Air Force Base, Dayton, OH USA; 4 Discovery Research, Abbott Nutrition, Columbus, OH USA; 5 Carl R. Woese Institute for Genomic Biology, University of Illinois, Champaign, IL USA; 6 Center for Brain Plasticity, University of Illinois, Urbana, IL USA; 7 Department of Psychology, University of Illinois, Urbana, IL USA; 8 Department of Bioengineering, University of Illinois, Champaign, IL USA; 9 Division of Nutritional Sciences, University of Illinois, Champaign, IL USA; 10 Neuroscience Program, University of Illinois, Champaign, IL USA

**Keywords:** Nutrition, Human behaviour, Physiology

## Abstract

Achieving military mission objectives requires high levels of performance from Airmen who operate under extreme physical and cognitive demands. Thus, there is a critical need to establish scientific interventions to enhance physical fitness and cognitive performance—promoting the resilience of Airmen and aiding in mission success. We therefore conducted a comprehensive, 12-week randomized controlled trial in active-duty Air Force Airmen (*n* = 148) to compare the efficacy of a multimodal intervention comprised of high-intensity interval aerobic fitness and strength training paired with a novel nutritional supplement [comprised of β-hydroxy β-methylbutyrate (HMB), lutein, phospholipids, DHA and selected micronutrients including B12 and folic acid] to high-intensity interval aerobic fitness and strength training paired with a standard of care placebo beverage. The exercise intervention alone improved several dimensions of physical fitness [strength and endurance (+ 8.3%), power (+ 0.85%), mobility and stability (+ 22%), heart rate (− 1.1%) and lean muscle mass (+ 1.4%)] and cognitive function [(episodic memory (+ 9.5%), processing efficiency (+ 7.5%), executive function reaction time (− 4.8%) and fluid intelligence accuracy (+ 19.5%)]. Relative to exercise training alone, the multimodal fitness and nutritional intervention further improved working memory (+ 9.0%), fluid intelligence reaction time (− 7.7%), processing efficiency (+ 1.8%), heart rate (− 2.4%) and lean muscle mass (+ 1.5%). These findings establish the efficacy of a multimodal intervention that incorporates aerobic fitness and strength training with a novel nutritional supplement to enhance military performance objectives and to provide optimal exercise training and nutritional support for the modern warfighter.

## Introduction

Adaptive military operations are a hallmark of modern warfare and depend on the capacity for flexible, resilient behavior. Airmen face extreme physical and mental demands that require the capacity to overcome the negative physical and psychological effects of operating in challenging field environments characterized by “volatile, uncertain, complex, and ambiguous” events^1^. A primary aim of research in the nutritional sciences is therefore to establish and validate nutritional interventions for the modern warfighter that are designed to provide optimal nutritional support and supplementation for body and mind. There is growing interest within the United States military to understand the effects of nutritional factors on warfighter resilience examined with respect to both physical and cognitive performance^2,3^.

Challenging field environments can prevent warfighters from realizing their optimal physical and cognitive performance. Participants with higher levels of stress and anxiety in military survival training exercises have lower scores on the Army Physical Readiness Test^4^. Acute stress in special operation soldiers may impair visuo-spatial capacity and working memory due to high dopamine and norepinephrine turnover in the prefrontal cortex, which is known to impair cognition and spatial working memory^5^. Surveys among military populations indicate that most warfighters use multivitamins, protein supplements or sports bars/drinks with a perception that these supplements improve their performance^6,7^. However, the efficacy of many of these supplements is not supported by randomized controlled trials^8,9^. Caffeine benefits executive function in sleep-deprived individuals; but the effects are acute and have negative side effects in some individuals^10^. Recent evidence further suggests that some supplements may contain dangerous or illegal substances that can have unknown consequences^11^. Thus, an enduring aim of research in the psychological and brain sciences is to establish nutritional supplements to enhance physical fitness and cognitive performance in active-duty military populations.

Accumulating evidence suggests that nutrients found in the Mediterranean diet promote brain and cognitive health. The Mediterranean diet includes olive oil as a source of monounsaturated fatty acids and polyphenols, fish and salmon that deliver phospholipids, omega-3 polyunsaturated fatty acids, lutein and vitamin D, and fruits and vegetables that provide antioxidants and vitamins B, C and E, carotenoids including lutein, folate, and polyphenols^12–16^. Elements of the Mediterranean diet are known to promote healthy vasculature by way of reducing total cholesterol and low-density lipoproteins (i.e., “bad cholesterol”)^17^, and improving endothelial function^18^. Broadly, these actions lower the risk of vascular comorbidities, dyslipidemia, hypertension, and coronary artery disease^19^. Within the brain, these actions are linked to a reduction in white matter lesions and promotion of white matter microstructure^19^. Furthermore, improvements in white matter brain structure are known to enhance cognitive performance (e.g., on measures of executive function and working memory^20^). Thus, combinations of these nutrients in the form of a novel nutritional supplement have the potential to improve the protective vascular, metabolic, antioxidant, and anti-inflammatory mechanisms promoted by individual nutrients and therefore to enhance brain function and cognition^13^.

Physical activity interventions improve cognition and the brain in multiple animal models, including rodents, dogs and monkeys^21^. Specifically, these models have demonstrated that exercise interventions result in (1) hippocampal neurogenesis; (2) increased synaptic activity in the brain; (3) growth of new blood vessels; (4) increased concentrations of brain derived neurotrophic factor (BDNF); (5) reduction of neurodegeneration; and (6) enhanced learning and memory^21^. The results from these animal models provide the basis for human studies of physical activity, fitness, and exercise. A meta-analysis of exercise interventions in humans revealed a moderate effect size for exercise leading to better cognition^22,23^. The meta-analysis showed that exercise improved a variety of cognitive tasks; but the largest effects were observed in tasks that engage the central executive network, including planning, problem solving, and working memory. Finally, the meta-analysis demonstrated that the combined effects of aerobic exercise, power and flexibility training had a greater cognitive benefit compared to only aerobic exercise training.

Motivated by these parallel lines of research, the present study examined the efficacy of a multi-modal fitness and nutritional intervention to enhance cognitive performance and physical fitness in United States Air Force Airmen. Specifically, we investigated: (1) whether a unimodal exercise training protocol enhanced fitness and cognition and (2) whether a multimodal fitness plus nutritional intervention resulted in fitness and cognitive gains beyond those conferred by the unimodal intervention.

## Methods

### Population

Study participants were active-duty Air Force Airmen recruited from Wright Patterson Air Force Base in Dayton, Ohio from January of 2016 through May of 2018. Study eligibility required participants to: (1) have active-duty status; (2) commit to study participation for 14 consecutive weeks; (3) be at least 18 but no older than 45 to minimize the risk of physical injury or cardiovascular occurrence due to the study’s required fitness activity; (4) not have a Department of Defense medical profile for mental and/or physical function limitation nor have a pregnancy profile; (5) not currently be taking prescription blood pressure medication; (6) cease taking herbal dietary supplements, performance supplements or any other substance that contained ingredients that might affect cardiovascular response with exercise one week before study participation began; (7) not have musculoskeletal injury that would limit their ability to engage in heavy resistance training and aerobic exercise; (8) not have cardiovascular or respiratory disease that would limit their ability to engage in heavy resistance training and aerobic exercise. 217 participants enrolled in the study; 205 began the intervention; and 148 completed the study. Only the data for those who completed the study were analyzed in this study and their demographics are presented in Table 1. Reasons for participants to drop (n = 70) include: (1) duty re-assignment or deployment; (2) muscle strain; and (3) attendance less than 80%; (4) not enough time.

**Table 1:** Demographics (n = 148).

Demographic	Supplement	Placebo	Total sample
Female	12%	16%	28%
Average age	30	30	30
Age SD	5.2	5.4	5.3
Age range	20–42	19–44	19–44
*Education*			
< 12 years	3%	3%	6%
12–16 years	32%	34%	66%
> 16 years	11%	16%	28%
*Rank*			
Airman First Class	6%	3%	9%
Staff Sergeant	11%	10%	21%
Technical Sergeant	8%	7%	15%
Major Sergeant	1%	4%	5%
Lieutenant	8%	11%	20%
Captain	9%	13%	22%
Major	2%	4%	6%
Colonel	1%	0%	21%

Percentages computed from total sample (n = 148).

*SD* standard deviation.

### Standard protocol approval, participant consent and recruitment

All experimental protocols were approved by the 711th Human Performance Wing Institutional Review Board (IRB). The 711th Human Performance Wing is part of the United States Air Force and comprises three units: the Airman Systems Directorate, US Air Force School of Aerospace Medicine and the Human Systems Integration Directorate^24^. Informed consent was obtained from all participants in this study. All methods were carried out in accordance with Air Force Research Lab regulations. Participants were recruited at Wright Patterson Air Force Base through IRB approved flyers and emails.

### Study design

Participants were randomly assigned to either the exercise plus nutritional supplement group (n = 70) or the exercise plus placebo group (n = 78). The sample size was based on a two-arm power analysis that specified an effect size of 0.20 and power of 0.90 and an attrition rate of 20%. Intervention assignment was counterbalanced for the 7 cohorts (about 25 participants per cohort) such that odd numbered cohorts received the supplement and even numbered cohorts received the placebo. Both experimenters and participants were blinded to group assignment. The exercise training intervention was identical for both groups. A battery of cognitive and physical fitness assessments was administered at pre- and post-intervention. This clinical trial was registered with the ISRCTN registry, number ISRCTN96342002, and was approved on July 28, 2020. This clinical trial was assigned a United States Air Force clearance of CLEARED on 28 Jul 2020 with case number MSC/PA-2020-0172.

### Exercise intervention

The exercise intervention was designed to enhance mission relevant strength, cardiovascular health and fitness. The training consisted of a total body resistance circuit designed to improve upper- and lower-body strength (Monday and Wednesday); a moderate cardiovascular workout designed to facilitate active recovery, flexibility and core strength (Tuesday and Thursday); and a cardiovascular endurance training and high intensity cardiovascular workout (Friday). Fitness training sessions were scheduled five days a week (Monday–Friday) for 12 weeks and session each lasted 45 minutes. Participants were guided on proper form for each exercise. Air Force Research Lab research staff, consisting of exercise physiologists, athletic trainers and strength coaches, designed and supervised the exercise intervention. All exercise sessions were supervised by at least one member of the research staff. Average attendance rate for the fitness training sessions was 90%. Supplementary Fig. 1, Supplementary Tables 1–5 and Supplementary Note 1 provide more details of the exercise training.

### Nutritional supplement and placebo intervention

The nutritional supplement beverage was designed to support both muscle and cognitive performance whereas the non-isocaloric standard of care placebo was designed to not promote physical or cognitive health. Both the supplement and placebo were ready-to-drink 8 fluid ounce liquid beverages, peach flavored and were packaged in identical plastic bottles to maintain blinding (Abbott Nutrition, Columbus, OH). The nutritional composition of each beverage is listed in Table 2. The nutritional supplement beverage is copyrighted by the University of Illinois Board of Trustees, 2020, and licensed under CC BY-NC 4.0 https://creativecommons.org/licenses/by-nc/4.0/.

**Table 2:** Nutritional composition of one 8-ounce placebo or nutritional supplement beverage.

Composition	Unit	Supplement	Placebo
Energy	Kcal	267	100
Protein	g	13	2
Carbohydrates	g	38	20
Fat	g	7	2
Ca-HMB	g	1.5	–
Choline	mg	100	–
DHA	mg	125	–
Folic acid	µg	100	–
Lutein	mg	6	–
Magnesium	g	0.1	–
Phospholipid	g	0.5	–
Selenium	µg	21	–
Vitamin B1	mg	0.4	–
Vitamin B2	mg	0.4	–
Vitamin B3	mg	5.0	–
Vitamin B5	mg	3.0	–
Vitamin B6	mg	0.8	–
Vitamin B12	µg	2	–
Vitamin C	mg	36	–
Vitamin D	IU	480	–
Vitamin E	mg	6.0	–
Zinc	mg	25	–

Participants were instructed to drink two 8-ounce servings a day for all 12 weeks of the intervention, for a total of 168 beverages throughout the study. Participants were advised to maintain their routine diet throughout the study and to drink one serving of the beverage 30 minutes before the fitness intervention and one serving 1 hour after the fitness intervention on Monday through Friday. On Saturday and Sunday, participants could consume the beverages at any time. The compliance rate for consuming the beverage was high. 47% of the participants consumed all 168 beverages; 51% of the participants consumed between 151 and 168 of the beverages; and 2% of the participants consumed between 141 and 151 beverages.

### Physical fitness assessment

A fitness battery was administered at pre- and post-intervention to assess the efficacy of the exercise training and nutritional supplement. Measurements were taken for 6 domains of physical health and fitness: (1) power (2) strength and endurance (3) mobility and stability (4) blood pressure (5) heart rate (6) lean muscle mass. Table 3 lists the fitness tests included in each domain along with a brief description of how they were measured.

**Table 3:** Tests administered for the physical fitness battery.

Fitness domain	Brief description
*Power*	
Abdominal circumference (inches)	Measure hip circumference using a tape measure
Sled push and pull R/L (seconds)	Push (15 yards) and pull (15 yards) a 140 pound sled twice
Rotation smash ball R/L (inches)	Distance thrown of a heavy medicine ball
Weight (pounds)	Measure body weight with scale
Wingate upper body (Watts/kg)	Arm crank with fixed resistance for 30 seconds
*Strength and endurance*	
Body fat (% body weight)	DEXA overall and segmented body composition
Modified Illinois agility (seconds)	Quickly complete a weaving running course
Pull ups	1 minute to complete as many as possible
Push ups	1 minute to complete as many as possible
Sit ups	1 minute to complete as many as possible
Standing long jump (inches)	Best of 3 jumps from a 2-foot static position
VO_2_ max (mL/kg/min)	Measure oxygen consumption on treadmill
Wingate lower body (Watts/kg)	Bicycle pedal with fixed resistance for 30 seconds
*Mobility and stability*	
Lateral bridge R/L (seconds)	Isometric hold until exhuastion
Lower Y balance test R/L	Multi-planar movement to test lower body balance
Supine bridge R/L (seconds)	Isometric hold until exhaustion
Upper Y balance test R/L	Multi-planar movement to test upper body balance
*Blood pressure*	
Diastolic blood pressure (mm Hg)	Average of 3 readings taken at rest
Systolic blood pressure (mm Hg)	Average of 3 readings taken at rest
*Heart rate*	
Maximum heart rate (beats/minute)	Number of beats/minute using electronic monitor
Resting heart rate (beats/minute)	Number of beats/minute using electronic monitor
*Anthropometrics*	
Height (inches)	Total body height
Leg length (inches)	Length of legs
*Lean muscle mass (pounds)*	DEXA overall and segmented body composition

‘R/L’ is an average of the right and left sides of the body.

### Cognitive assessment

A cognitive battery was administered at pre- and post-intervention to assess the efficacy of the exercise training and nutritional supplement. Measurements were taken for 6 domains of cognitive function: (1) short term memory (2) episodic memory (3) fluid intelligence (4) working memory (5) executive function and (6) processing efficiency and reaction time. Table 4 lists the cognitive tests included in each domain along with a brief description of how they were measured. Supplementary Note 2 provides further detail for each cognitive test.

**Table 4:** Tests administered for the cognitive battery.

Cognitive domain	Brief description
*Short term memory*	
Immediate free recall (IFR) words	Recall as many previously seen words as possible
Immediate free recall (IFR) pictures	Recall as many previously seen pictures as possible
Keep track	Recall the last instance of a given category from a previously seen set of words
*Episodic memory*	
Paired associates (PA) immediate recall	Given one word from a pair of words previously seen, recall the other word immediately
Paired associates (PA) delayed recall	Given one word from a pair of words previously seen, recall the other word after a delay
*Fluid Intelligence*	
Number series accuracy (Acc)	Identify the pattern of numbers
Letter series accuracy (Acc)	Identify the pattern of letters
*Working memory*	
Symmetry span	Hold items in memory while checking symmetry in a matrix
Rotation span	Hold items in memory while rotating letters
*Executive function*	
Stroop congruent accuracy (Acc)	Determine if the font color and word color match
Stroop incongruent accuracy (Acc)	Determine if the font color and word color mismatch
*Processing efficiency*	
Symbol digit modalities	Use a lookup table to translate symbols into numbers
*Reaction time (RT)*	
Number series, letter series, stroop congruent and incongruent	The amount of time to respond to each item

### Biomarker assessment

Blood plasma samples were collected at pre- and post-intervention and analyzed for vitamin B12^25^, folate^26^, triglycerides^27^, high-density lipoprotein (HDL), ferritin^28^, cortisol^29^ and several fatty acids (trans, saturated, mono-unsaturated, omega-3 and omega-6 polyunsaturated)^30^. Lutein was measured in the eye with Macular Pigment Optical Density (MPOD).

### Statistical analysis

#### Statistical software

All data analyses were conducted using the R Studio interface (Version 1.0.143), which runs on the base R installation (Version 3.5.1^31,32^). The R packages *psych*, *effsize*, *compute.es*, *lsr* and *BayesFactor* were used for the analyses in this manuscript^33–37^.

#### Statistical models

Principal component analysis, or PCA, was used to identify common structure in the physical fitness and cognitive test batteries. A component was retained if its eigenvalue was greater than 1.0. The number of components retained accounted for approximately 75% of the variance. A variable was included in a component if the loading had a magnitude with an absolute value of 0.50 or greater. Once the components were identified, composite scores were formed by averaging together variables which comprised each component from the PCA. The composite scores were computed separately for pre- and post-intervention. Composite scores reduce the number of analyses and reduce the likelihood of committing a Type 1 error.

One-way paired t-tests assessed differences between pre- and post-intervention measures within intervention groups. The ANOVA model was used to test for difference at pre-intervention between groups. The ANCOVA model tests for post-intervention differences across groups, while controlling for baseline performance.

#### Model evidence

*p* values for the statistical models provide evidence for an improvement from pre- to post-intervention if they are less than the threshold of 0.05. Cohen’s *d* measures the magnitude of the effect size from pre- to post-intervention. A Cohen’s d value of 0.0 ≤ *d* < 0.01 is a very small effect size, 0.01 ≤ *d* < 0.20 is a small effect size, 0.20 ≤ *d* < 0.50 is a medium effect size, 0.50 ≤ *d* < 0.80 is a large effect size, 0.80 ≤ *d* < 1.20 is a very large effect size and *d* ≥ 1.20 is huge^38,39^. Bayes factors, *Bf*, quantify the likelihood of a change from pre- to post-intervention. A *Bf* < 1 means there is no change from pre- to post-intervention; 1 ≤ *Bf* < 3 is some evidence for a change from pre- to post-intervention; 3 ≤ *Bf* < 20 is positive evidence of a change from pre- to post-intervention; 20 ≤ *Bf* < 150 is strong evidence of a change from pre- to post-intervention; and *Bf* > 150 is decisive evidence for a change from pre- to post-intervention^40^.

## Results

### Checking for random assignment

Because participants were randomly assigned to intervention groups, differences in demographics, fitness or cognitive test measures at pre-intervention were not expected. Table 1 presents the demographic composition of the participants, including Air Force Rank. The sample was 28% female; had an average age of 30; and had an educational composition with 6% not completing high school, 66% completing some college and 28% completing graduate or professional schooling. Demographics did not differ between the exercise plus supplement and exercise plus placebo groups (see Supplementary Note 3 for statistical results). Supplementary Tables 6 and 7 present the means and standard deviations of all physical fitness measures and cognitive tests included in the pre-intervention test battery. Pre-intervention means did not differ between the exercise plus supplement and exercise plus placebo intervention for 41 out of 42 fitness and cognitive measures (see Supplementary Note 3 for statistical results). The similarity between the exercise plus supplement and exercise plus placebo groups for both demographic composition and pre-intervention fitness and cognitive scores provides strong evidence that participants were randomly assigned into groups.

### Principal component analysis

Principal Component Analysis (PCA) was used to identify the underlying structure of the fitness and cognitive batteries. For fitness, 21 variables from Table 3 were included in PCA and five components accounted for 77% of the variance. The component names, also listed in Table 3 are Power, Strength and Endurance, Mobility and Stability, Blood Pressure and Heart Rate. Supplementary Table 8 presents the loadings for each component. For cognition, 11 variables from Table 4 were included in PCA and five components accounted for 72% of the variance. The component names, also listed in Table 4 are Short Term Memory, Episodic Memory, Fluid Intelligence, Working Memory and Executive Function. Supplementary Table 9 presents the loadings for each component. Supplementary Note 4 presents further details of the PCA.

### Efficacy of the exercise training plus placebo intervention

Table 5 reports the statistical results providing evidence for changes in physical fitness, cognition and biomarkers associated with the exercise training plus placebo intervention. Overall, 61% (14 out of 23) of the assessment metrics changed. Five of the six physical fitness measures improved from pre- to post-intervention: power, strength and endurance, mobility and stability, heart rate and lean muscle mass. Blood pressure did not improve. Four of the eight cognitive measures improved from pre- to post-intervention in the exercise training plus placebo intervention: episodic memory, fluid intelligence accuracy, executive function reaction time and processing efficiency. Cognitive measures that did not improve include short term memory, executive function accuracy, fluid intelligence reaction time and working memory. Five of the biomarkers decreased in concentration (cortisol, ferritin, folate, vitamin B12 and low-density lipoprotein) while nine biomarkers did not change (lutein density in the fovea or parafovea, HDL, triglyceride levels and all five fatty acids). Supplementary Tables 10–12 report the pre- and post-intervention means and standard deviations for the physical fitness and cognitive batteries and biomarkers for the exercise training plus placebo group.

**Table 5:** Statistical results for the efficacy of the exercise plus placebo intervention.

	t-stat	*df*	*p* value	*d*	*Bf*
*Physical fitness*					
Power*	3.22	73	0.001	0.37	28.1
Strength and endurance*	12.3	65	10^−16^	1.52	10^16^
Mobility and stability*	5.53	74	10^−7^	0.64	10^6^
Blood pressure	− 0.87	76	0.19	0.10	0.29
Heart rate*	− 2.16	75	0.018	0.25	2.2
Lean muscle mass*	3.42	76	0.001	0.39	48.9
*Cognition*					
Short term memory	− 2.4	72	0.99	0.29	0.04
Episodic memory*	2.4	55	0.01	0.32	3.65
Fluid intelligence acc*	5.5	73	10^−7^	0.64	10^5^
Fluid intelligence RT	0.55	72	0.71	0.06	0.09
Working memory	− 1.64	73	0.95	0.19	0.05
Executive function acc	− 1.14	73	0.87	0.13	0.06
Executive function RT*	− 3.2	73	0.0011	0.37	23.2
Processing efficiency*	2.6	73	0.005	0.23	6.4
*Biomarkers*					
Cortisol (µg/dL)*	− 1.60	77	0.056	0.18	0.80
Ferritin (ng/mL)*	− 6.58	76	10^−9^	0.75	10^6^
Folate (ng/mL)*	− 1.65	76	0.051	0.19	0.87
High density lipoprotein (mg/dL)	0.77	77	0.22	0.09	0.26
Low density lipoprotein (mg/dL)*	− 2.33	77	0.01	0.26	3.14
Lutein density in fovea	− 0.13	30	0.55	0.02	0.17
Lutein density in parafovea	− 0.43	30	0.66	0.08	0.14
Triglycerides (mg/dL)	− 0.19	76	0.58	0.02	0.11
Vitamin B12 (pg/mL)*	− 3.36	76	0.001	0.38	40.6
Saturated fatty acids (mol%)	0.30	77	0.38	0.04	0.16
Monounsaturated fatty acids (mol%)	1.25	77	0.11	0.14	0.47
Omega-3 PUFAs (mol%)	− 0.43	77	0.67	0.05	0.18
Omega-6 PUFAs (mol%)	− 0.51	77	0.69	0.06	0.09
Trans fatty acids (mol%)	− 1.53	77	0.06	0.17	0.71

t-stat is the value of the 1-sided t-statistic (where negative values represent decreases from pre- to post-intervention); *df* is the degrees of freedom of the t-test; *p* value is the frequentist *p* value of the t-test; *d* is Cohen’s *d*; and *Bf* is the Bayes factor for the t-test. An * in the row header indicates the exercise plus placebo intervention modified that indicator. PUFA stands for polyunsaturated fatty acid.

### Efficacy of the exercise training plus supplement intervention

Table 6 reports the statistical results providing evidence for changes in physical fitness, cognition and biomarkers associated with the exercise training plus supplement. Overall, 83% (19 out of 23) of the assessment metrics changed. All six physical fitness measures improved from pre- to post-intervention: power, strength and endurance, mobility and stability, heart rate, blood pressure and lean muscle mass. Six of the eight cognitive measures improved in the exercise training plus nutritional supplement intervention: episodic memory, fluid intelligence accuracy and reaction time, working memory, executive function reaction time and processing efficiency. Accuracy for short term memory and executive function did not improve. Eight of the biomarkers changed (cortisol, ferritin, omega-6 polyunsaturated fats and low-density lipoprotein decreased; folate, vitamin B12, omega-3 polyunsaturated fats and lutein density in the fovea all increased). Follow-up analyses of the omega-3 fatty acids revealed that serum concentrations of both docosahexaenoic (DHA) and eicosapentaenoic (EPA) increased but docosapentaenoic (DPA) decreased. For omega-6 fatty acids, follow-up analyses revealed concentrations of arachadonic, adrenic, and docosapentaenoic acid all decreased. Six biomarkers did not change (HDL, triglycerides, saturated fatty acids, trans fatty acids, mono-unsaturated fatty acids and lutein density in the parafovea). Supplementary Tables 10–12 report the pre- and post-intervention means and standard deviations for the physical fitness and cognitive batteries and biomarkers for the exercise training plus supplement group.

**Table 6:** Statistical results for the efficacy of the exercise plus supplement intervention.

	t-stat	*df*	*p* value	*d*	*Bf*
*Physical fitness*					
Power*	4.97	63	10^−6^	0.62	6324
Strength and endurance*	10.9	50	10^−15^	1.52	10^12^
Mobility and stability*	6.1	69	10^−8^	0.73	10^6^
Blood pressure*	− 2.7	69	0.004	0.33	7.9
Heart rate*	− 6.2	68	10^−8^	0.75	10^6^
Lean muscle mass*	9.2	69	10^−14^	1.10	10^10^
*Cognition*					
Short term memory	− 2.2	63	0.98	0.27	0.05
Episodic memory*	2.28	58	0.013	0.30	3.05
Fluid intelligence acc*	5.3	65	10^−7^	0.65	10^4^
Fluid intelligence RT*	− 1.85	65	0.034	0.23	1.30
Working memory*	1.81	67	0.04	0.22	1.20
Executive function acc	− 4.3	66	0.001	0.52	0.03
Executive function RT*	− 5.6	66	10^−7^	0.68	10^4^
Processing efficiency*	5.4	69	10^−7^	0.47	10^4^
*Biomarkers*					
Cortisol (µg/dL)*	− 1.61	54	0.057	0.22	0.92
Ferritin (ng/mL)*	− 5.96	54	10^−8^	0.80	10^5^
Folate (ng/mL)*	1.74	54	0.043	0.24	1.15
High density lipoprotein (mg/dL)	1.04	54	0.15	0.14	0.41
Low density lipoprotein (mg/dL)*	− 2.54	54	0.007	0.35	5.45
Lutein density in fovea*	1.62	13	0.06	0.43	1.42
Lutein density in parafovea	1.07	13	0.15	0.29	0.73
Triglycerides (mg/dL)	1.25	54	0.11	0.17	0.54
Vitamin B12 (pg/mL)*	2.02	54	0.024	0.27	1.89
Saturated fatty acids (mol%)	0.24	55	0.41	0.04	0.18
Monounsaturated fatty acids (mol%)	− 0.15	55	0.56	0.02	0.13
Omega-3 PUFAs (mol%)*	6.72	55	10^−9^	0.90	10^6^
Omega-6 PUFAs (mol%)*	− 3.52	55	0.00043	0.47	62.2
Trans fatty acids (mol%)	− 0.86	55	0.20	0.11	0.33

t-stat is the value of the 1-sided t-statistic (where negative values represent decreases from pre- to post-intervention); *df* is the degrees of freedom of the t-test; *p* value is the frequentist *p* value of the t-test; *d* is Cohen’s *d*; and *Bf* is the Bayes factor for the t-test. An * in the row header indicates the exercise plus placebo intervention modified that indicator. PUFA stands for polyunsaturated fatty acid.

### Efficacy of the unimodal versus the multimodal intervention

**Figure 1:**
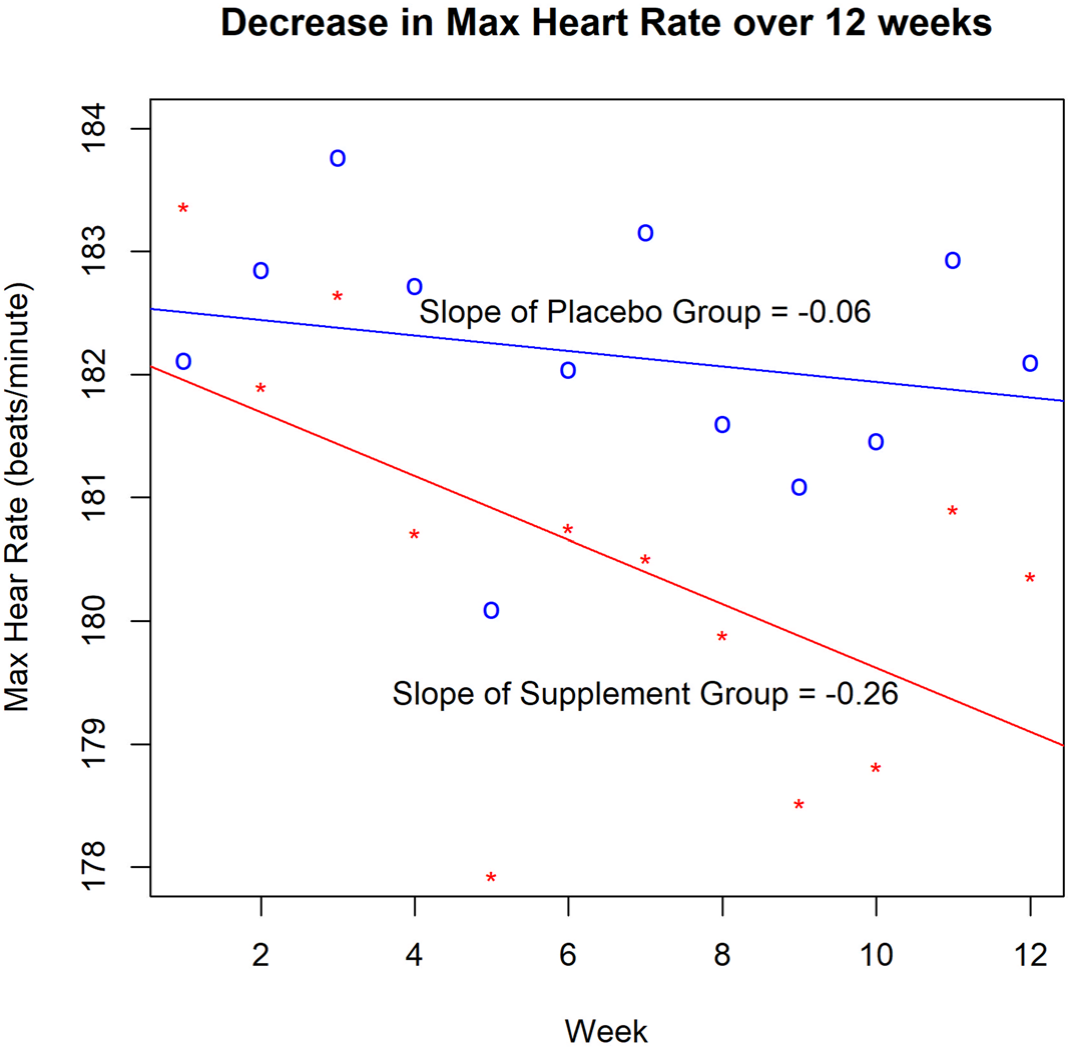
Maximum heart rate for the exercise plus supplement (red line) and exercise plus placebo (blue line) interventions. The x-axis is the week of the study and the y-axis is the average maximum heart rate. Each point on the plot represents the average maximum heart rate across all participants in that intervention condition for that week.

The unimodal exercise plus placebo intervention was compared to the multimodal exercise plus supplement using an ANCOVA model to determine if the addition of the nutritional supplement conferred incremental physical and cognitive benefits. Measures were only tested in the ANCOVA if there was a change from pre- to post-intervention in Table 5 or 6. The multimodal exercise plus supplement resulted in further improvement beyond those observed for the exercise plus placebo intervention for heart rate, lean muscle mass, fluid intelligence reaction time, working memory, processing efficiency, folate, vitamin B12, omega-3 and omega-6 polyunsaturated fatty acids (Table 7). Figure 1 plots the average maximum heart rate for the multimodal versus the unimodal intervention group and demonstrates a steeper rate of decline in maximum heart rate for the nutritional supplement. For the remaining measures in Table 7 (power, strength and endurance, mobility and stability, blood pressure, episodic memory, fluid intelligence accuracy, executive function reaction time, cortisol and ferritin levels and lutein density in the fovea) the multimodal exercise plus supplement did not confer incremental benefits beyond the unimodal exercise plus placebo. Supplementary Table 13 provides the mean values underlying the statistical tests in Table 7. Supplementary Table 14 presents Bayes factor ratios comparing the two interventions to provide a quantitative measure of their relative efficacy, which provides further evidence of the benefit of the nutritional supplement. Supplementary Note 5 provides further discussion of Supplementary Table 14.

**Table 7:** Statistical results comparing the efficacy of the unimodal exercise plus placebo intervention to the multimodal exercise plus supplement intervention.

	F-stat	*df*	*p* value	*d*	*Bf*
*Physical fitness*					
Power	1.46	(2,135)	0.23	0.20	0.35
Strength and endurance	1.80	(2,114)	0.18	0.22	0.48
Mobility and stability	0.05	(2,142)	0.82	0.04	0.18
Blood pressure	0.75	(2,144)	0.39	0.14	0.48
Heart rate*	7.42	(2,142)	0.007	0.45	7.03
Lean muscle mass*	13.9	(2,144)	0.0003	0.61	63.4
*Cognition*					
Episodic memory	1.77	(2,115)	0.19	0.22	0.14
Fluid intelligence acc	0.27	(2,140)	0.60	0.09	0.20
Fluid intelligence RT*	2.28	(2,139)	0.13	0.25	2.09
Working memory*	6.81	(2,142)	0.01	0.43	8.27
Executive function RT	0.99	(2,141)	0.32	0.16	0.53
Processing efficiency*	3.12	(2,141)	0.08	0.29	1.07
*Biomarkers*					
Cortisol (µg/dL)	0.04	(2,129)	0.84	0.03	0.21
Low density lipoprotein (mg/dL)	0.13	(2,129)	0.72	0.06	0.21
Ferritin (ng/mL)	0.30	(2,129)	0.59	0.09	0.27
Folate (ng/mL)*	14.6	(2,129)	0.0002	0.66	2.65
Lutein density in fovea	0.48	(2,42)	0.49	0.12	0.49
Vitamin B12 (pg/mL)*	16.5	(2,129)	10^−5^	0.70	79.9
Omega-3 PUFAs (mol%)*	46.1	(2,131)	10^−16^	1.18	10^7^
Omega-6 PUFAs (mol%)*	6.7	(2,131)	0.011	0.45	5.93

F-stat is the value of the ANCOVA model which included group as a factor and pre-intervention performance as a covariate; *df* is the degrees of freedom of the ANCOVA model; *p* value is the frequentist *p* value of the ANCOVA model; *d* is Cohen’s *d*; and *Bf* is the Bayes factor for the ANCOVA. An * in the row header means the exercise plus supplement intervention improved that indicator more than the exercise plus placebo intervention. To minimize the number of analyses conducted—and the resulting Type 1 error—Table 7 only includes rows from Table 5 or 6 that had a significant result. PUFA stands for polyunsaturated fatty acid. Supplementary Table 13 lists the means underlying these statistical tests.

## Discussion

We conducted an RCT in healthy, fit and active duty Air Force servicemen and women (n = 148) to investigate the efficacy of an exercise training intervention and a novel nutritional supplement for improving physical fitness and cognition. Exercise training interventions are known to improve overall physical health and cognition and we also observed these improvements in the current study^41^. Our study extends this body of work by demonstrating that the combination of a nutritional supplement and an exercise training intervention provided physical health and cognitive gains beyond those from exercise alone. The large sample size of the present study and the large number of assessments across physical fitness, cognition and biomarkers permit five primary conclusions.

First, both interventions improved measures of physical fitness. The exercise training plus placebo intervention improved 21 individual measures of physical fitness and the exercise training plus nutritional supplement intervention improved 23 individual measures of physical fitness. Given the huge Cohen’s *d* effect sizes in some cases, and the decisive *Bf* results, there is clear and compelling evidence that the exercise training intervention was effective and that it improved physical fitness.

Second, both interventions improved measures of cognition. The exercise training plus placebo intervention improved 8 individual measures of cognition. The exercise training plus nutritional supplement intervention effectively improved 11 individual measures of cognition. These results suggest that the combination of a nutritional supplement with the exercise training intervention selectively extended the benefits of the exercise training plus placebo intervention for some of the cognitive outcomes. Consistent with previous research^41^, the exercise training plus placebo intervention drove improvements in cognitive function.

The third primary conclusion from the study is that the multimodal exercise training plus nutritional supplement intervention conferred advantages on physical fitness and cognition beyond the effects of the unimodal exercise training plus placebo intervention. Heart rate, lean muscle mass, fluid intelligence reaction time, working memory and processing efficiency all improved more for the multimodal exercise training plus nutritional supplement. Broadly, this is consistent with recent studies demonstrating advantages for multimodal interventions, relative to unimodal interventions, for improving physical fitness or cognition^42,43^.

Fourth, changes in biomarkers provide further evidence to support the efficacy of the interventions. One inflammatory marker, ferritin, decreased for both intervention groups; but the nutritional supplement did not reduce ferritin levels more than the exercise training intervention. Increasingly, ferritin is recognized as an important marker of inflammation and cell damage; higher levels are associated with worse cognitive functioning^44^. In the current study, the exercise intervention decreased inflammation, as measured by ferritin. Vitamin B12, folate, the omega-3 polyunsaturated fatty acid docosahexaenoic acid (DHA) and lutein were consumed daily as part of the nutritional supplement intervention. Increased serum concentrations of these four nutrients were observed in the exercise training and nutritional supplement intervention only. Concentrations of vitamin B12 and folate decreased in the exercise training plus placebo intervention. Concentrations of omega-6 polyunsaturated fatty-acids, which were not part of the nutritional supplement, decreased only for the exercise plus nutritional supplement intervention. Importantly, concentrations of trans, saturated and monounsaturated fatty acids did not change from pre- to post-intervention for either intervention group. Because neither the nutritional formula nor the placebo contained any of these biomarkers, we observe selectivity of biological uptake for the biomarkers that were included in the nutritional formula.

These changes in serum concentrations of biomarkers in participants in the nutritional supplement intervention suggest possible mechanisms that might have driven improvements in cognition and physical fitness. B vitamins are critical for brain health^45^. Younger adult populations show stronger associations between B vitamin supplementation and cognitive improvement^46^. Therefore, it is reasonable to conclude that B vitamins from the nutritional supplement in the current study are partially responsible for some of the observed improvements in cognitive function in the exercise training plus nutritional supplement group. The nutritional supplement intervention group had increased concentrations of both docosahexaenoic acid (DHA) and eicosapentaenoic acid (EPA). Long chain polyunsaturated fatty acids, or PUFAs, like DHA and EPA, are found in high concentrations in the brain’s gray matter. PUFAs in the brain are known to promote neuronal growth and synapse formation and facilitate neural transmission^47^. Long chain omega-6 polyunsaturated fatty acids are important for brain and cognitive health; but this effect is most pronounced when they are in a balanced ratio with the omega-3 polyunsaturated fats^48,49^. Long chain omega-3 and omega-6 fatty acids compete for chemical conversion to various structures and molecules inside and outside cells. In the current study, for the nutritional intervention group, omega-6 concentrations for arachadonic, adrenic, and docosapentaenoic acids all decreased but the omega-3 concentrations of DHA and EPA increased. The increased availability of omega-3’s from the nutritional supplement resulted in a reduction in the ratio of omega-6/omega-3 from 10:1 at the start of the study to 8:1 by the end of the intervention. The ratio remained unchanged at 10:1 from pre- to post-intervention for the exercise plus placebo group. The observed reduction in concentration of several omega-6 fatty acids in the nutritional supplement group was most likely due to the increased availability of omega-3 fatty acids from the nutritional supplement, thereby driving the smaller omega-6/omega-3 ratio which, in turn, contributed to the improved cognitive outcomes for the group receiving the nutritional supplement.

Protein, HMB (β-hydroxy β-methylbutyrate) and vitamin D, all of which were included in the nutritional supplement, increase muscle protein synthesis and muscle growth and improve physical fitness. Protein supplementation increases muscle mass and muscle strength^50^. Furthermore, a dose-dependent relationship exists between the amount of dietary protein and uptake into the body for overall whole-body protein levels and incorporation into muscle fibers^51^. β-hydroxy β-methylbutyrate (HMB), which was included in the nutritional supplement, synergistically works with protein for increasing lean muscle mass and improves recovery in resistance exercise^52,53^. Vitamin D facilitates absorption of calcium in bones and supports muscle growth^54^. Enhancements to overall physical fitness and health emerged when protein, HMB, protein and vitamin D were taken together as part of an oral nutritional supplement^55^. And the effects of protein supplementation can be augmented by exercise. Interventions that combine exercise training with nutritional supplements that promote muscle recovery and growth provide the body with necessary building blocks for greater fitness training gains^56–58^.

Although the current study provides evidence that the nutritional supplement improved physical fitness and cognition when coupled with exercise training, more research is needed to refine these findings. First, the present study only considered the efficacy of the intervention at the group level. Having demonstrated group level changes to the interventions, follow-up studies should seek to understand the role of individual variability. There is a growing body of research demonstrating that different individuals may need a different number of intervention sessions to achieve the same gain^59^. And different individuals start interventions with different levels of physical fitness and cognitive ability. Accounting for these differential starting points, and possibly using baseline information as a predictor of the possible training gains, could allow further tailoring of intervention protocols and sensitivity of testing to achieve optimal performance gains for individuals. Second, it is not known if interventions like exercise training and a nutritional supplement beverage have upper limits to the physical fitness or cognitive gains achievable, whether at the group or individual level. Dose–response curves would help tease this out. Third, the current study used principal component analysis (PCA) to aggregate physical fitness, cognitive and biomarker measures according to common structure or function. Part of the motivation for using PCA was to amplify the underlying signal and reduce the number of statistical tests required; but there are other statistical approaches which could derive further insights about the efficacy of the interventions. Fourth, it was not possible to include a true control condition because active duty military populations necessarily undergo a certain amount of physical movement in their day-to-day activity. However, Supplementary Note 6 provides the results of an analysis including a passive control group from another study, where study participants matched the demographic and physiological profile of the current study, and who also completed the same cognitive battery as participants in the current study. These results are consistent with the results reported in the current study that did not include a true control group. Fifth, the mechanism of improvement for cognition from the addition of the nutritional supplement needs to be elucidated. There are at least 3 possibilities: (a) the supplement enhanced fitness, which in turn improved cognition more than the exercise training intervention alone, (b) the supplement directly enhanced cognition, (c) the supplement improved fitness, which improved cognition and the supplement also directly improved cognition. Functional and structural brain imaging analyses would facilitate an understanding of how the brain changes in an intervention, as would a study with intervention groups designed to isolate this mechanism. Sixth, more research needs to be conducted to further understand the mechanism of some biomarker results. Serum B vitamin concentrations increased for the nutritional supplement group but decreased for the placebo group. Hence, the nutritional formula restored the B vitamins depleted through exercise. Future research should examine whether increased B vitamin concentration is partially responsible for the gains in cognition observed in the nutritional supplement intervention. Finally, because the nutritional supplement is a liquid that needs to be refrigerated, its use would be limited to garrison or home settings. However, because the nutritional supplement appears to enhance the body and brain with continued usage, it should not be considered to aid immediate safety or performance, as might be required in a field environment.

The nutritional supplement was a user-friendly way to ensure each participant received equivalent amounts of scientifically proven daily allowances of both macro- and micro-nutrients that supported health benefits. A study using a whole food intervention might confer some advantages over a nutritional supplement, such as greater bioavailability and adsorption of key nutrients; but running a 3-month RCT with whole food intervention presents several challenges. First, the whole food intervention would need to select a variety of foods but still ensure equivalent dosage of key nutrients. Second, in the current study, the non-isocaloric placebo represented a normal diet since those participants had no dietary restrictions. In a whole food intervention, it would be harder to have a true placebo. Third, administering whole food meals is costly (both in terms of time and money) and challenging to track consumption. In the current study, it was easy to watch participants drink two 8-ounce servings of the liquid nutritional supplement in the lab every day; but tracking the consumption of whole food meals is much more challenging. Despite these challenges, future work should attempt to implement a nutritional intervention that includes an isocaloric control. Moreover, researchers should seek to develop nutritional supplements that more closely approximate whole food. To this end, some researchers have begun to elucidate how patterns of nutrient biomarkers, which presumably relate to eating a healthy, whole food diet, enhance cognitive and brain function^60,61^.

In conclusion, the current study supports the efficacy of a multimodal fitness and nutritional intervention to improve both physical fitness and cognition in Air Force Airmen. Moreover, these changes in the body and brain align with corresponding changes in blood-based biomarkers of nutrition. Our study also demonstrates a wider range of improvements and larger effect sizes due to multimodal training compared to unimodal fitness training alone. While the findings are based on a large sample of Air Force Airmen and demonstrate that physical fitness and cognitive enhancement is achievable, the multimodal lifestyle intervention documented in this study could easily be implemented in other real-world contexts to optimize human performance.

## Supplementary information

Supplementary Information.

## Data Availability

The individual de-identified participant data and related study documents can be made available upon request.

## Code availability

All analyses were conducted in R using standard packages and functions (see "Methods" section).
