# Author Correction: Enhanced physical and cognitive performance in active duty Airmen: evidence from a randomized multimodal physical fitness and nutritional intervention

**DOI:** 10.1038/s41598-021-81800-9

**Published:** 2021-02-09

**Authors:** Christopher E. Zwilling, Adam Strang, Evan Anderson, Jennifer Jurcsisn, Erica Johnson, Tapas Das, Matthew J. Kuchan, Aron K. Barbey

**Affiliations:** 1 Decision Neuroscience Laboratory, University of Illinois, 405 North Mathews Avenue, Urbana, IL 61801 USA; 2 Beckman Institute for Advanced Science and Technology, University of Illinois, Urbana, IL USA; 3 Applied Neuroscience Branch, Wright Patterson Air Force Base, Dayton, OH USA; 4 Discovery Research, Abbott Nutrition, Columbus, OH USA; 5 Carl R. Woese Institute for Genomic Biology, University of Illinois, Champaign, IL USA; 6 Center for Brain Plasticity, University of Illinois, Urbana, IL USA; 7 Department of Psychology, University of Illinois, Urbana, IL USA; 8 Department of Bioengineering, University of Illinois, Champaign, IL USA; 9 Division of Nutritional Sciences, University of Illinois, Champaign, IL USA; 10 Neuroscience Program, University of Illinois, Champaign, IL USA

Correction to: *Scientific Reports* https://doi.org/10.1038/s41598-020-74140-7, published online 19 October 2020.

The Supplementary Information file that accompanies this Article contained errors in Supplementary Table 13, where values were rounded incorrectly.

In the column ‘Exercise + Supplement Pre’, in the row ‘Blood Pressure’

“102 (10.4)”

now reads:

“102.0 (10.4)”

In the column ‘Exercise + Supplement Pre’, in the row ‘Heart Rate’

“127 (10.2)”

now reads:

“127.2 (10.2)”

In the column ‘Exercise + Supplement Pre’, in the row ‘Lean Muscle Mass (pounds)’

“123 (22.7)”

now reads:

“123.1 (22.7)”

In the column ‘Exercise + Supplement Pre’, in the row ‘Fluid Intelligence RT’

“514 (114)”

now reads:

“514.5 (114)”

In the column ‘Exercise + Supplement Pre’, in the row ‘Executive Function RT’

“695 (133)”

now reads:

“693.5 (133)”

In the same Supplementary Table 13, in the column ‘Exercise + Supplement Post’, in the row ‘Heart Rate’

“122 (9.6)”

now reads:

“122.6 (9.6)”

In the column ‘Exercise + Supplement Post’, in the row ‘Lean Muscle Mass (pounds)’

“126 (22.8)”

now reads:

“126.5 (22.8)”

In the column ‘Exercise + Supplement Post’, in the row ‘Fluid Intelligence RT’

“493 (97.1)”

now reads:

“491.4 (97.1)”

In the column ‘Exercise + Supplement Post’, in the row ‘Executive Function RT’

“639 (124)”

now reads:

“638.5 (124)”

In the same Supplementary Table 13, in the column ‘Exercise + Placebo Pre’, in the row ‘Heart Rate’

“126 (9.6)”

now reads:

“126.1 (9.6)”

In the column ‘Exercise + Placebo Pre’, in the row ‘Lean Muscle Mass (pounds)’

“123 (23.2)”

now reads:

“122.8 (23.2)”

In the column ‘Exercise + Placebo Pre’, in the row ‘Fluid Intelligence RT’

“496 (111)”

now reads:

“496.4 (111)”

In the column ‘Exercise + Placebo Pre’, in the row ‘Executive Function RT’

“684 (136)”

now reads:

“683.6 (136)”

Finally, in the same Supplementary Table 13, in the column ‘Exercise + Placebo Post’, in the row ‘Heart Rate’

“125 (9.7)”

now reads:

“124.4 (9.7)”

In the column ‘Exercise + Placebo Post’, in the row ‘Lean Muscle Mass (pounds)’

“124 (22.1)”

now reads:

“124.2 (22.1)”

In the column ‘Exercise + Placebo Post’, in the row ‘Fluid Intelligence RT’

“505 (82)”

now reads:

“504.8 (82)”

In the column ‘Exercise + Placebo Post’, in the row ‘Executive Function RT’

“643 (143)”

now reads:

“643.8 (143)”

Additionally, in the legend of Supplementary Table 13 the formulas for computing the values in the Abstract were omitted,

“RT is reaction time in milliseconds.”

now reads:

“RT is reaction time in milliseconds. The values in this table were used to derive the values in the abstract of the manuscript. Exercise effects reported in the abstract were derived by computing the percent change for the Exercise + Placebo group: (Exercise + Placebo Post—Exercise + Placebo Pre)/Exercise + Placebo Pre. Multimodal fitness and nutritional intervention effects reported in the abstract were derived by computing a relative difference percent change: [(Exercise + Supplement Post—Exercise + Supplement Pre)/Exercise + Supplement Pre]-[(Exercise + Placebo Post—Exercise + Placebo Pre)/Exercise + Placebo Pre].”

The original version of Supplementary Table 13 appears below as Table 1.

**Table 1:** The original version of Supplementary Table 13.

Composite	Exercise + Supplement Pre	Exercise + Supplement Post	Exercise + Placebo Pre	Exercise + Placebo Post
Power	75.6 (15.3)	76.9 (15.6)	76.0 (16.7)	76.8 (16.0)
Strength and endurance	36.2 (8.5)	38.7 (8.7)	35.9 (9.4)	38.7 (9.0)
Mobility and stability	34.1 (13.1)	40.4 (15.8)	33.3 (13.9)	39.4 (15.9)
Blood pressure	102 (10.4)	99.4 (9.5)	99.8 (10.9)	99.1 (10.7)
Heart rate	127 (10.2)	122 (9.6)	126 (9.6)	125 (9.7)
Lean muscle mass (pounds)	123 (22.7)	126 (22.8)	123 (23.2)	124 (22.1)
Short term memory	30.0 (5.2)	29.2 (5.5)	30.3 (5.5)	29.4 (6.1)
Episodic memory	2.62 (1.87)	3.16 (2.37)	3.31 (2.19)	3.97 (2.31)
Fluid intelligence accuracy	8.46 (1.95)	9.32 (1.96)	8.27 (2.32)	9.18 (1.84)
Fluid intelligence RT	514 (114)	493 (97.1)	496 (111)	505 (82)
Working memory	15.9 (6.5)	17.0 (7.5)	16.3 (6.9)	15.6 (6.6)
Executive function accuracy	0.98 (0.03)	0.96 (0.05)	0.97 (0.03)	0.96 (0.10)
Executive function RT	695 (133)	639 (124)	684 (136)	643 (143)
Processing efficiency	53.1 (9.0)	57.8 (11.0)	52.7 (11.2)	55.1 (10.3)

As a result of the errors in Supplementary Table 13, in the Abstract,

“The exercise intervention alone improved several dimensions of physical fitness [strength and endurance (+ 8.3%), power (+ 0.85%), mobility and stability (+ 22%), heart rate (− 1.1%) and lean muscle mass (+ 1.4%)] and cognitive function [(episodic memory (+ 9.5%), processing efficiency (+ 7.5%), executive function reaction time (− 4.8%) and fluid intelligence accuracy (+ 19.5%)]. Relative to exercise training alone, the multimodal fitness and nutritional intervention further improved working memory (+ 9.0%), fluid intelligence reaction time (− 7.7%), processing efficiency (+ 1.8%), heart rate (− 2.4%) and lean muscle mass (+ 1.5%).”

now reads:

“The exercise intervention alone improved several dimensions of physical fitness [strength and endurance (+ 7.8%), power (+ 1.1%), mobility and stability (+ 18.3%), heart rate (− 1.3%) and lean muscle mass (+ 1.1%)] and cognitive function [(episodic memory (+ 19.9%), processing efficiency (+ 4.6%), executive function reaction time (− 5.8%) and fluid intelligence accuracy (+ 11.0%)]. Relative to exercise training alone, the multimodal fitness and nutritional intervention further improved working memory (+ 11.2%), fluid intelligence reaction time (− 6.2%), processing efficiency (+ 4.3%), heart rate (− 2.3%) and lean muscle mass (+ 1.6%).”

These errors have now been corrected in the PDF and HTML versions of the Article, and in the Supplementary Information file that accompanies the Article.

