# Supplementary material for: Enhanced physical and cognitive performance in active duty Airmen: evidence from a randomized multimodal physical fitness and nutritional intervention
Source: Sci Rep. 2020 Oct 19;10:17826. doi: 10.1038/s41598-020-74140-7 (PMC7572526; doi:10.1038/s41598-020-74140-7)
Supplement: Supplementary file 1 — Supplementary Information. [file 41598_2020_74140_MOESM1_ESM.docx]

**Enhanced Physical and Cognitive Performance in Active Duty Airmen: Evidence from a Randomized Multimodal Physical Fitness and Nutritional Intervention**

Christopher E. Zwilling^1,2^, Adam Strang^3^, Evan Anderson^1,2^, Jennifer Jurcsisn^3^, Erica Johnson^3^, Tapas Das^4^, Matthew J. Kuchan^4^, and Aron K. Barbey^1,2,5,6,7,8,9,10^

^1^ Decision Neuroscience Laboratory, University of Illinois, Urbana, IL. USA

^2^ Beckman Institute for Advanced Science and Technology, University of Illinois, Urbana, IL. USA

^3^ Applied Neuroscience Branch, Wright Patterson Air Force Base, Dayton, OH, USA

^4^ Discovery Research, Abbott Nutrition, Columbus, OH, USA

^5^ Carl R. Woese Institute for Genomic Biology, University of Illinois, Champaign, IL. USA

^6^ Center for Brain Plasticity, University of Illinois, Urbana, IL. USA

^7^ Department of Psychology, University of Illinois, Urbana, IL. USA

^8^ Department of Bioengineering, University of Illinois, Champaign, IL. USA

^9^ Division of Nutritional Sciences, University of Illinois, Champaign, IL. USA

^10^ Neuroscience Program, University of Illinois, Champaign, IL. USA

**Address for correspondence**

Decision Neuroscience Laboratory

Beckman Institute for Advanced Science and Technology

University of Illinois at Urbana Champaign

405 North Mathews Avenue

Urbana, IL 61801

Email: [barbey@illinois.edu](mailto:barbey@illinois.edu) (AKB)

Web: <http://DecisionNeuroscienceLab.org>

**Supplementary Materials.**

**Supplementary Figure 1. Display of real-time heart rate monitoring.**


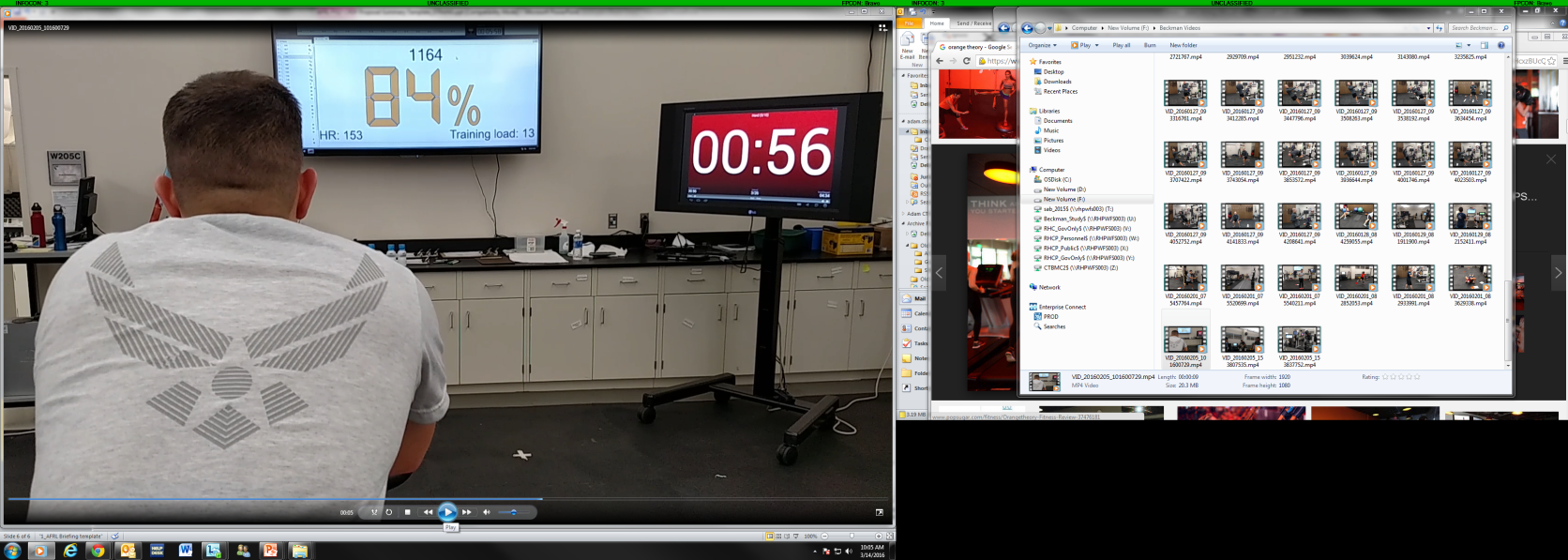


Heart rate percentage maximum (displayed on white screen) and time spent above heart rate threshold (displayed on red screen). These screens were updated in real-time during all circuit and cardiovascular workouts.

**Supplementary Table 1. Schedule of exercise training.**

|  | Monday | Tuesday | Wednesday | Thursday | Friday |
| --- | --- | --- | --- | --- | --- |
| Weeks of the Intervention | Total Body Resistance | Active Recovery & Light Cardio | Total Body Resistance | Active Recovery & Light Cardio | Intense Cardio |
| 2, 6 & 10 | Circuit 1 | Circuit 1 | Circuit 2 | Circuit 2 | Circuit 1 |
| 3, 7 & 11 | Circuit 3 | Circuit 3 | Circuit 4 | Circuit 4 | Circuit 2 |
| 4, 8 & 12 | Circuit 5 | Circuit 5 | Circuit 6 | Circuit 6 | Circuit 3 |
| 5, 9 & 13 | Circuit 7 | Circuit 7 | Circuit 8 | Circuit 8 | Circuit 4 |

**Supplementary Table 2. Exercise category and sequence for the metabolic resistance circuits and examples of unique circuits used in this study.**

| Exercise Category and Sequence | Circuit 1 workout | Circuit 2 workout |
| --- | --- | --- |
| Anterior Chain-Lower Body | Diagonal overhead dumbbell squat (10x each side) | Side to side squat (30x) |
| Posterior Chain – Lower Body | Staggered 2-Leg Roman Dead Lift (KB) (12 reps) | Retro Lunge with Back Kick (10x each side) |
| Agility/Speed | zigzag ladder (2 laps) | Anterior/Lateral Bounding (24x) |
| Whole Body | Lunge & Twist (24 lunges) | Cable Turn and Pull Up (12x) |
| Whole Body | Sled pull/TRX row (1 Lap) | Sled push (1 Lap) |
| Whole Body | Cable Turn and Pull Down (12x) | Landmine hop & twist (24x) |
| Push – Upper Body | Suspension Band Push-up (max reps) | Staggered-hands push-up (max rep) |
| Agility/Speed | Jump 180s (20x) | In-and-Out ladder (2 laps) |
| Pull – Upper Body | Wide Grip pull-up (max reps) | Suspension Band inverted Row (20x) |

**Supplementary Table 3. Moderate intensity workouts and % Max HR thresholds.**

| Moderate Intensity Workout (22 min) | % Max HR | Duration |
| --- | --- | --- |
| Warm-up | Below 60% | 2 min |
| Moderate | 70-80% | 5min |
| Easy | 60-70% | 1 min |
| Moderate | 70-80% | 5 min |
| Easy | 60-70% | 1 min |
| Moderate | 70-80% | 5 min |
| Easy | 60-70% | 1 min |
| Cool Down | Below 60% | 2 min |

**Supplementary Table 4. Hight intensity workouts and % Max HR thresholds.**

| High Intensity Interval Workout (35 min, 30 sec) | % Max HR | Duration |
| --- | --- | --- |
| Warm-up | Below 60% | 2 min |
| Moderate | 70-80% | 2min, 30 sec. |
| Hard | 90-100% | 1 min |
| Easy | 60-70% | 1 min |
| Transition | - | 45 sec. |
| Moderate | 70-80% | 2min, 30 sec. |
| Hard | 90-100% | 1 min |
| Easy | 60-70% | 1 min |
| Transition | - | 45 sec. |
| Moderate | 70-80% | 2min, 30 sec. |
| Hard | 90-100% | 1 min |
| Easy | 60-70% | 1 min |
| Transition | - | 45 sec. |
| Moderate | 70-80% | 2min, 30 sec. |
| Hard | 90-100% | 1 min |
| Easy | 60-70% | 1 min |
| Transition | - | 45 sec. |
| Moderate | 70-80% | 2min, 30 sec. |
| Hard | 90-100% | 1 min |
| Easy | 60-70% | 1 min |
| Cool Down | Below 60% | 2 min |

**Supplementary Table 5. Moderate workout core exercises.**

| Exercise Category | Core Circuit 1 | Core Circuit 2 |
| --- | --- | --- |
| Lateral abdominals | Side Plank (30 sec. each side) | Side Crunch (15x each side) |
| Anterior abdominals | Scissors (16x) | Sphinx Plank (30 sec) |
| Low back muscles | Bird Dog (10x each side) | Superman (12x, 4 sec. hold) |
| Anterior Abdominals | Heel touch (20x each side) | Swiss Ball Knee Tucks (15x) |

**Supplementary Table 6.** Means and standard deviations for pre-intervention fitness measures for the exercise plus supplement and exercise plus placebo.

| Fitness Domain | Exercise + Supplement | Exercise +  Placebo |
| --- | --- | --- |
| **Power** |  |  |
| Abdominal Circumference (inches) | 34.5 (3.3) | 34.5 (3.8) |
| Sled Rope Pull R/L (seconds) | 10.7 (3.2) | 11.2 (4.5) |
| Sled Push & Pull R/L (seconds) | 32.1 (9.1) | 33.3 (9.2) |
| Rotation Smash Ball R/L (inches) | 147 (38.6) | 148 (43.4) |
| Weight (pounds) | 178 (29.2) | 179 (31.8) |
| Wingate Upper Body (Watts/kg) | 2.9 (0.9) | 2.8 (0.9) |
| **Strength & Endurance** |  |  |
| Body Fat (% body weight) | 28.6 (6.9) | 28.9 (7.8) |
| Modified Illinois Agility (seconds) | 20.4 (2.3) | 20.8 (2.3) |
| Pull Ups | 16.6 (8.9) | 16.7 (9.2) |
| Push Ups | 47.7 (15.5) | 46.3 (16.0) |
| Sit Ups | 49.2 (11.6) | 46.6 (11.5) |
| Standing Long Jump (inches) | 77.6 (13.1) | 76.4 (16.3) |
| VO2 Max (mL/kg/min) | 43.7 (8.7) | 45.5 (10.7) |
| Wingate Lower Body (Watts/kg) | 5.8 (1.3) | 5.7 (1.2) |
| **Mobility & Stability** |  |  |
| Lateral Bridge R/L (seconds) | 60.5 (20.5) | 60.2 (22.7) |
| Lower Y Balance Test R/L | 0.99 (0.07) | 1.01 (0.10) |
| Supine Bridge R/L (seconds) | 74.0 (31.8) | 70.9 (32.8) |
| Upper Y Balance Test R/L | 0.89 (0.09) | 0.88 (0.11) |
| **Blood Pressure** |  |  |
| Diastolic Blood Pressure (mm Hg) | 77.4 (9.1) | 75.8 (8.8) |
| Systolic Blood Pressure (mm Hg) | 127 (11.7) | 124 (13.0) |
| **Heart Rate** |  |  |
| Maximum Heart Rate (beats/minute) | 183 (9.6) | 182 (8.8) |
| Resting Heart Rate (beats/minute) | 71.2 (10.9) | 70.3 (10.4) |
| **Anthropometrics** |  |  |
| Height (inches) | 68.2 (3.4) | 68.4 (3.5) |
| Leg Length (inches) | 35.7 (2.5) | 35.2 (2.3) |
| **Lean Muscle Mass (pounds)** | 123 (22.7) | 123 (23.2) |

‘R/L’ is an average of the right and left sides of the body.

**Supplementary Table 7.** Means and standard deviations for pre-intervention cognitive measures for the exercise plus supplement and exercise plus placebo.

| Cognitive Domain | Exercise + Supplement | Exercise +  Placebo |
| --- | --- | --- |
| **Short Term Memory** |  |  |
| IFR Words Pre | 23.5 (4.5) | 24.1 (5.6) |
| IFR Picture Pre | 27.0 (4.8) | 26.7 (5.0) |
| Keep Track Pre | 39.6 (6.2) | 39.9 (5.9) |
| **Episodic Memory** |  |  |
| PA Immediate Pre | 2.58 (1.85) | 3.40 (2.22) |
| PA Delayed Pre | 2.68 (1.89) | 3.22 (2.15) |
| **Fluid Intelligence** |  |  |
| Number Series Pre | 5.73 (1.72) | 5.87 (2.03) |
| Number Series RT Pre | 518 (130) | 488 (127) |
| Letter Series Pre | 11.2 (2.19) | 10.7 (2.61) |
| Letter Series RT Pre | 512 (97) | 503 (95) |
| **Working Memory** |  |  |
| Symmetry Span Pre | 18.8 (6.9) | 19.1 (7.6) |
| Rotation Span Pre | 12.9 (6.2) | 13.6 (6.2) |
| **Executive Function** |  |  |
| Stroop Congruent Acc Pre | 0.986 (0.02) | 0.984 (0.02) |
| Stroop Congruent RT Pre | 641 (113) | 629 (106) |
| Stroop Incongruent Acc Pre | 0.968 (0.04) | 0.958 (0.04) |
| Stroop Incongruent RT Pre | 749 (153) | 738 (166) |
| **Processing Efficiency** |  |  |
| Symbol Digit Modalities Pre | 53.1 (8.97) | 52.7 (11.2) |

Rows represent number correct, unless RT (reaction time, milliseconds) or Acc (Accuracy) is included. IFR is Immediate Free Recall and PA is Paired Associates. ‘Pre’ is pre-intervention and ‘Post’ is post-intervention.

**Supplementary Table 8.** PCA results for the physical fitness battery.

|  | Power | Strength & Endurance | Mobility & Stability | BP | HR |
| --- | --- | --- | --- | --- | --- |
| Ab Circumference | 0.69 |  |  |  |  |
| Lean Muscle Mass | 0.86 |  |  |  |  |
| Sled Rope Pull R/L | -0.85 |  |  |  |  |
| Sled Push & Pull R/L | -0.84 |  |  |  |  |
| Rotation Smash Ball R/L | 0.81 |  |  |  |  |
| Weight | 0.83 |  |  |  |  |
| Wingate Upper Body R/L | 0.73 |  |  |  |  |
| Body Fat |  | -0.88 |  |  |  |
| Modified Illinois Agility |  | -0.58 |  |  |  |
| Pull Ups |  | 0.63 |  |  |  |
| Push Ups |  | 0.72 |  |  |  |
| Sit Ups |  | 0.77 |  |  |  |
| Standing Long Jump |  | 0.63 |  |  |  |
| VO2 Max |  | 0.74 |  |  |  |
| Wingate Lower Body R/L |  | 0.68 |  |  |  |
| Lateral Bridge R/L |  |  | 0.67 |  |  |
| Lower Y Balance Test R/L |  |  | 0.75 |  |  |
| Supine Bridge R/L |  |  | 0.69 |  |  |
| Upper Y Balance Test R/L |  |  | 0.80 |  |  |
| Diastolic Blood Pressure |  |  |  | 0.87 |  |
| Systolic Blood Pressure |  |  |  | 0.67 |  |
| Maximum Heart Rate |  |  |  |  | 0.85 |
| Resting Heart Rate |  |  |  |  | 0.48 |
| Proportion Variance | 27% | 25% | 12% | 7% | 6% |
| Cumulative Variance | 27% | 52% | 64% | 72% | 77% |

‘BP’ is Blood Pressure; ‘HR’ is Heart Rate; ‘R/L’ is an average of the right and left arms, legs or sides of the body. Only the largest loading for each fitness measure is displayed.

**Supplementary Table 9.** PCA results for the cognitive battery.

|  | STM | EM | Gf | WM | EF |
| --- | --- | --- | --- | --- | --- |
| Immediate Free Recall Words | 0.82 |  |  |  |  |
| Immediate Free Recall Pictures | 0.78 |  |  |  |  |
| Keep Track Words Recalled | 0.48 |  |  |  |  |
| Paired Associates Words Recalled (Immediate) |  | 0.80 |  |  |  |
| Paired Associates Words Recalled (Delay) |  | 0.92 |  |  |  |
| Number Series |  |  | 0.75 |  |  |
| Letter Sets |  |  | 0.81 |  |  |
| Rotation Span |  |  |  | 0.89 |  |
| Symmetry Span |  |  |  | 0.76 |  |
| Stroop Congruous |  |  |  |  | 0.82 |
| Stroop Incongruous |  |  |  |  | 0.81 |
| Proportion Variance | 16% | 15% | 14% | 14% | 13% |
| Cumulative Variance | 16% | 32% | 46% | 59% | 72% |

STM is Short Term Memory; EM is Episodic Memory; Gf is fluid intelligence; WM is working memory; and EF is executive function.

**Supplementary Table 10.** Means and standard deviations for pre- and post-intervention fitness measures for the exercise plus supplement and exercise plus placebo at pre- and post-intervention.

| Fitness Domain | Exercise +  Supplement  Pre | Exercise + Supplement  Post | Exercise + Placebo  Pre | Exercise + Placebo  Post |
| --- | --- | --- | --- | --- |
| **Power** |  |  |  |  |
| Abdominal Circumference (inches) | 34.5 (3.3) | 34.1 (3.5) | 34.5 (3.8) | 34.2 (3.5) |
| Sled Rope Pull R/L (seconds) | 10.7 (3.2) | 9.3 (2.4) | 11.2 (4.5) | 9.8 (3.0) |
| Sled Push & Pull R/L (seconds) | 32.1 (9.1) | 29.8 (9.1) | 33.3 (9.2) | 31.0 (7.4) |
| Rotation Smash Ball R/L (inches) | 147 (38.6) | 155 (41.0) | 148 (43.4) | 157 (44.5) |
| Weight (pounds) | 178 (29.2) | 180 (29.3) | 179 (31.8) | 179 (30.9) |
| Wingate Upper Body R/L (Watts/kg) | 2.9 (0.9) | 3.1 (0.8) | 2.8 (0.9) | 3.1 (0.9) |
| **Strength & Endurance** |  |  |  |  |
| Body Fat (% body weight) | 28.6 (6.9) | 27.3 (6.9) | 28.9 (7.8) | 27.9 (7.7) |
| Modified Illinois Agility (seconds) | 20.4 (2.3) | 19.7 (2.1) | 20.8 (2.3) | 20.2 (2.1) |
| Pull Ups | 16.6 (8.9) | 21.9 (10.7) | 16.7 (9.2) | 21.7 (10.1) |
| Push Ups | 47.7 (15.5) | 51.5 (14.1) | 46.3 (16.0) | 51.7 (15.9) |
| Sit Ups | 49.2 (11.6) | 53.6 (10.7) | 46.6 (11.5) | 52.4 (10.1) |
| Standing Long Jump (inches) | 77.6 (13.1) | 80.2 (12.8) | 76.4 (16.3) | 80.2 (14.7) |
| VO2 Max (mL/kg/min) | 43.7 (8.7) | 49.3 (11.1) | 45.5 (10.7) | 49.8 (10.5) |
| Wingate Lower Body R/L (Watts/kg) | 5.8 (1.3) | 6.3 (1.1) | 5.7 (1.2) | 6.1 (1.2) |
| **Mobility & Stability** |  |  |  |  |
| Lateral Bridge R/L (seconds) | 60.5 (20.5) | 72.7 (25.2) | 60.2 (22.7) | 72.3 (24.5) |
| Lower Y Balance Test R/L | 0.99 (0.07) | 1.03 (0.07) | 1.01 (0.10) | 1.05 (0.09) |
| Supine Bridge R/L (seconds) | 74.0 (31.8) | 86.8 (37.8) | 70.9 (32.8) | 83.1 (39.0) |
| Upper Y Balance Test R/L | 0.89 (0.09) | 0.93 (0.10) | 0.88 (0.11) | 0.93 (0.10) |
| **Blood Pressure** |  |  |  |  |
| Diastolic Blood Pressure (mm Hg) | 77.4 (9.1) | 74.4 (7.7) | 75.8 (8.8) | 73.9 (8.4) |
| Systolic Blood Pressure (mm Hg) | 127 (11.7) | 124 (11.3) | 124 (13.0) | 124 (13.1) |
| **Heart Rate** |  |  |  |  |
| Maximum Heart Rate (beats/minute) | 183 (9.6) | 180 (8.7) | 182 (8.8) | 182 (8.3) |
| Resting Heart Rate (beats/minute) | 71.2 (10.9) | 64.8 (10.5) | 70.3 (10.4) | 66.9 (11.0) |
| **Lean Muscle Mass (pounds)** | 123 (22.7) | 126 (22.8) | 123 (23.2) | 124 (22.1) |
| **Anthropometrics (Pre only)** |  |  |  |  |
| Height (inches) | 68.2 (3.4) | -- | 68.4 (3.5) | -- |
| Leg Length (inches) | 35.7 (2.5) | -- | 35.2 (2.3) | -- |

‘R/L’ is an average of the right and left side of the body.

**Supplementary Table 11.** Means and standard deviations for pre- and post-intervention cognitive measures for the exercise plus supplement and exercise plus placebo at pre- and post-intervention.

| Cognitive Domain | Exercise + Supplement | Exercise + Placebo |
| --- | --- | --- |
| **Short Term Memory** |  |  |
| IFR Words Pre | 23.5 (4.5) | 24.1 (5.6) |
| IFR Words Post | 22.8 (5.8) | 22.8 (5.8) |
| IFR Picture Pre | 27.0 (4.8) | 26.7 (5.0) |
| IFR Pictures Post | 26.8 (4.3) | 27.6 (5.4) |
| Keep Track Pre | 39.6 (6.2) | 39.9 (5.9) |
| Keep Track Post | 38.1 (6.5) | 37.8 (7.0) |
| **Episodic Memory** |  |  |
| PA Immediate Pre | 2.58 (1.85) | 3.40 (2.22) |
| PA Immediate Post | 3.15 (2.35) | 4.00 (2.25) |
| PA Delayed Pre | 2.68 (1.89) | 3.22 (2.15) |
| PA Delayed Post | 3.16 (2.38) | 3.94 (2.36) |
| **Fluid Intelligence** |  |  |
| Number Series Pre | 5.73 (1.72) | 5.87 (2.03) |
| Number Series Post | 7.67 (2.00) | 7.68 (1.92) |
| Number Series RT Pre | 518 (130) | 488 (127) |
| Number Series RT Post | 487 (105) | 507 (88) |
| Letter Series Pre | 11.2 (2.19) | 10.7 (2.61) |
| Letter Series Post | 11.0 (1.92) | 10.7 (1.75) |
| Letter Series RT Pre | 512 (97) | 503 (95) |
| Letter Series RT Post | 499 (90) | 503 (76) |
| **Working Memory** |  |  |
| Symmetry Span Pre | 18.8 (6.9) | 19.1 (7.6) |
| Symmetry Span Post | 20.2 (7.4) | 18.8 (6.9) |
| Rotation Span Pre | 12.9 (6.2) | 13.6 (6.2) |
| Rotation Span Post | 13.7 (7.6) | 12.5 (6.3) |
| **Executive Function** |  |  |
| Stroop Congruent Acc Pre | 0.986 (0.02) | 0.984 (0.02) |
| Stroop Congruent Acc Post | 0.971 (0.03) | 0.969 (0.08) |
| Stroop Congruent RT Pre | 641 (113) | 629 (106) |
| Stroop Congruent RT Post | 602 (107) | 605 (107) |
| Stroop Incongruent Acc Pre | 0.968 (0.04) | 0.958 (0.04) |
| Stroop Incongruent Acc Post | 0.950 (0.06) | 0.950 (0.12) |
| Stroop Incongruent RT Pre | 749 (153) | 738 (166) |
| Stroop Incongruent RT Post | 675 (140) | 682 (179) |
| **Processing Efficiency** |  |  |
| Symbol Digit Modalities Pre | 53.1 (8.97) | 52.7 (11.2) |
| Symbol Digit Modalities Post | 57.8 (11.0) | 55.1 (10.3) |

Rows represent number correct, unless RT (reaction time, milliseconds) or Acc (Accuracy) is included. IFR is Immediate Free Recall and PA is Paired Associates. ‘Pre’ is pre-intervention and ‘Post’ is post-intervention. PA Immediate Pre differed between the intervention groups.

**Supplementary Table 12**. Means and standard deviations for pre- and post-intervention biomarker measures for the exercise plus supplement and exercise plus placebo at pre- and post-intervention.

| Biomarker | Supplement  Pre | Supplement  Post | Placebo  Pre | Placebo  Post |
| --- | --- | --- | --- | --- |
| Cortisol ug/dL | 10.9 (4.61) | 9.6 (4.10) | 10.5 (4.3) | 9.7 (3.5) |
| Ferritin (ng/mL) | 119 (64.5) | 84.3 (54.4) | 110 (86.4) | 81.5 (63.0) |
| Folate (ng/mL) | 15.1 (4.48) | 16.0 (3.40) | 13.9 (4.69) | 13.1 (4.59) |
| High Density Lipoprotein (mg/dL) | 51.6 (13.3) | 53.5 (16.6) | 49.3 (13.5) | 49.9 (13.8) |
| Low Density Lipoprotein (mg/dL) | 113 (32.2) | 107 (29.7) | 102 (27.0) | 97.8 (24.0) |
| Lutein density in fovea | 25.3 (3.2) | 26.2 (3.6) | 26.3 (2.6) | 26.1 (2.7) |
| Lutein density in parafovea | 28.8 (2.6) | 29.3 (2.1) | 28.8 (2.3) | 29.1 (2.7) |
| Triglycerides (mg/dL) | 119 (70.6) | 134 (78.2) | 114 (62.5) | 117 (70.4) |
| Vitamin B12 (pg/mL) | 593 (194) | 643 (203) | 604 (266) | 545 (235) |
| Saturated Fatty Acids (mol %) | 38.5 (1.3) | 38.5 (1.3) | 38.5 (1.2) | 38.6 (1.3) |
| Monounsaturated Fatty Acids (mol%) | 12.2 (1.2) | 12.1 (1.0) | 12.2 (1.2) | 12.3 (1.2) |
| Omega-3 PUFA (mol %) | 4.6 (1.1) | 5.7 (1.3) | 4.9 (1.8) | 4.9 (1.6) |
| Omega-6 PUFA (mol %) | 43.9 (2.2) | 42.8 (2.2) | 43.5 (2.0) | 43.4 (2.0) |
| Trans Fatty Acids (mol %) | 0.82 (0.18) | 0.80 (0.22) | 0.87 (0.25) | 0.82 (0.25) |

PUFA is polyunsaturated fatty acid.

**Supplementary Table 13**. Composite means and standard deviations for pre- and post-intervention fitness and cognitive components for the exercise plus supplement and exercise plus placebo at pre- and post-intervention. Each composite represents the average for the individual measures in Supplementary Tables 6 and 7.

| Composite | Exercise +  Supplement  Pre | Exercise + Supplement  Post | Exercise + Placebo  Pre | Exercise + Placebo  Post |
| --- | --- | --- | --- | --- |
| **Power** | 75.6 (15.3) | 76.9 (15.6) | 76.0 (16.7) | 76.8 (16.0) |
| **Strength & Endurance** | 36.2 (8.5) | 38.7 (8.7) | 35.9 (9.4) | 38.7 (9.0) |
| **Mobility & Stability** | 34.1 (13.1) | 40.4 (15.8) | 33.3 (13.9) | 39.4 (15.9) |
| **Blood Pressure** | 102 (10.4) | 99.4 (9.5) | 99.8 (10.9) | 99.1 (10.7) |
| **Heart Rate** | 127 (10.2) | 122 (9.6) | 126 (9.6) | 125 (9.7) |
| **Lean Muscle Mass (pounds)** | 123 (22.7) | 126 (22.8) | 123 (23.2) | 124 (22.1) |
| **Short Term Memory** | 30.0 (5.2) | 29.2 (5.5) | 30.3 (5.5) | 29.4 (6.1) |
| **Episodic Memory** | 2.62 (1.87) | 3.16 (2.37) | 3.31 (2.19) | 3.97 (2.31) |
| **Fluid Intelligence Accuracy** | 8.46 (1.95) | 9.32 (1.96) | 8.27 (2.32) | 9.18 (1.84) |
| **Fluid Intelligence RT** | 514 (114) | 493 (97.1) | 496 (111) | 505 (82) |
| **Working Memory** | 15.9 (6.5) | 17.0 (7.5) | 16.3 (6.9) | 15.6 (6.6) |
| **Executive Function Accuracy** | 0.98 (0.03) | 0.96 (0.05) | 0.97 (0.03) | 0.96 (0.10) |
| **Executive Function RT** | 695 (133) | 639 (124) | 684 (136) | 643 (143) |
| **Processing Efficiency** | 53.1 (9.0) | 57.8 (11.0) | 52.7 (11.2) | 55.1 (10.3) |

RT is reaction time in milliseconds.

**Supplementary Table 14.** Bayes factors for the exercise plus placebo, exercise plus supplement and their ratio.

|  | Exercise+  Placebo | Exercise + Supplement | Ratio | Ratio |
| --- | --- | --- | --- | --- |
| Domain | *Bf* | *Bf* | EP:ES | ES:EP |
| **Physical Fitness** |  |  |  |  |
| Power | 28.1 | 6324 | -- | 225 |
| Strength & Endurance | 10^16^ | 10^12^ | 5738 | -- |
| Mobility & Stability | 10^6^ | 10^6^ | -- | 8.9 |
| Blood Pressure | 0.29 | 7.9 | -- | 27.2 |
| Heart Rate | 2.2 | 10^6^ | -- | 10^5^ |
| Lean Muscle Mass | 48.9 | 10^12^ | -- | 10^10^ |
| **Cognition** |  |  |  |  |
| Short Term Memory | 0.04 | 0.05 | * | * |
| Episodic Memory | 3.65 | 3.05 | 1.2 | -- |
| Fluid Intelligence Acc | 59291 | 19290 | 3.1 | -- |
| Fluid Intelligence RT | 0.09 | 1.30 | -- | 14.8 |
| Working Memory | 0.05 | 1.20 | -- | 38.1 |
| Executive Function Acc | 0.06 | 0.03 | * | * |
| Executive Function RT | 23.2 | 57154 | -- | 1409 |
| Processing Efficiency | 6.4 | 34375 | -- | 5371 |

‘EP’ is the exercise plus placebo intervention and ‘ES’ is the exercise plus supplement intervention. *Bf* is the Bayes factor. ‘Acc’ is accuracy and ‘RT’ is reaction time. An * in the Ratio columns means the Bayes factors ratios are not worth interpreting because there is no evidence that either intervention in those cases improved the cognitive measure (i.e. the Bayes factor are less than 1.0). The Ratio columns are derived by dividing the *Bf* of one intervention (FS or FP) by the *Bf* of the other intervention (FS or FP). A dashed line -- in the Ratio columns means the value is less than 1.

**Supplementary Note 1. Exercise Intervention.**

Supplementary Table 1 shows the type of workout each day of each week and which circuit was completed on each of the 12 weeks of the intervention.

Metabolic resistance circuits, which were completed on Mondays and Wednesdays, included nine exercises performed in repeated succession for a duration of 45 minutes. These workouts were designed to improve whole-body muscle endurance, coordination, and agility/speed (see Supplementary Tables 1 and 2 for more details). Exercises chosen in the study design: 1) supported the mission demands of ‘most’ Air Force career fields; 2) were time-efficient; 3) would facilitate performance on the Air Force Fitness Test which favors a lean body profile and muscular endurance.

The intensity and volume of circuit workouts was determined by each Airman’s own willingness and capability to increase their resistance (in consultation with the supervising strength coaches and athletic trainers) and individual heart rate (HR) dynamics. HR was monitored and displayed in real-time using Polar Team ® during all workouts (see Supplementary Figure 1). This feedback was used during resistance circuits to dynamically set and adjust rest intervals according to an Airman’s own cardiovascular fitness.

An Airman was not permitted to move on to the next exercise in the circuit until their HR had returned to 60-70% of their maximum HR (Max HR). Max HR was obtained at baseline during the VO2 max test and then adjusted over time as new HR Max’s were identified during workouts. This rule had two important consequences. First, it put a governor on workout intensity, since 60-70% of Max HR corresponds to an aerobic energy state (below ventilatory threshold) for most Airmen. This approach prevented overemphasis/concentration on anaerobic energy during metabolic circuits, which is posited to result in overtraining and/or injury^1^. Second, as the fitness of the Airmen improved over time, HR recovery during rest intervals became faster. This caused the rest intervals themselves to become shorter, with the consequence being that total exercise volume (e.g., total number of exercises completed) increased throughout the intervention.

The intensities of both the moderate resistance circuits, which were completed on Tuesdays and Thursdays (see Supplementary Tables 1 and 3 for more details), and high intensity cardiovascular workouts completed on Fridays (see Supplementary Tables 1 and 4 for more details) also relied on the use of real-time HR feedback. Specifically, Airmen were given a HR goal for each interval within these workouts, which corresponded to a percentage of their Max HR. This approach adapts training recommendations of an endurance coach^2^.

This design approach ensured that the relative intensity and volume of each cardiovascular workout was similar across all Airmen, whereas the absolute intensity was governed by each Airman’s own ability. Similar to the circuit workouts, the result was control over the proportion of time each Airmen spent in different energy states (aerobic vs. anaerobic) throughout each session.

It is important to note that for the moderate cardiovascular workouts Airmen chose a single exercise modality (i.e., bike, elliptical or treadmill) for each session, with the caveat that an Airman could not choose the treadmill more than once per week. For high intensity workouts, Airmen rotated between all three modalities during the transition periods lasting 45 seconds. These rules were imposed to reduce the likelihood of overuse injuries caused by ambulatory activities, which are prominent in military populations^3^.

Finally, a short series of core exercises were included at the end of each moderate cardiovascular workout session. These exercises, presented in Supplementary Table 5, were designed improve the endurance strength of anterior/lateral abdominal and low back musculature, which facilitate performance in most exercises included in this intervention.

This combination of unique exercise design approaches not only likely contributed to the broad improvements in physical fitness observed in the manuscript, but also resulted in a low injury rate (7 Airmen, representing less than 5% of the sample). Of these injuries, six were acute muscle strains and one was a fall. To put this rate into context, the Air Force recently reported a 12.5% injury rate for those going through basic training^4^. And this rate is lower than that reported from other services.

**Supplementary Note 2. Cognitive assessment.**

The cognitive battery encompassed tests from 5 domains: short term memory, episodic memory, fluid intelligence, working memory and executive function. A description of the tests used within each domain are presented below.

*Short term memory.* Short term memory was assessed with three tasks: Immediate Free Recall (IFR) pictures, IFR Words and Keep Track. In IFR (pictures or words), the participant is shown a sequence of 12 images (all objects, no “action” images) or words (all 4 letter nouns/verbs), 750 ms for each stimuli^5-7^. For the picture recall, the participant has 30 seconds to type out at least one word for every image they recall from the trial. Each image has multiple words that could be accepted as a correct response (i.e. if shown an image of a chimpanzee, answers “chimpanzee” and “monkey” would both be acceptable). For the word recall, the participant has 30 seconds to recall as many words as possible. There are 5 test trials for pictures and 5 test trials for words. The Keep Track task measures subjects’ ability to update items held in short-term memory^8^. In the task, a series of word stimuli are presented, each belonging to a category. Subjects must remember only the last item presented from each category. The example figure below shows a list of stimuli presented on the left side of the figure (stimuli were presented sequentially) and the right side of the figure shows an example of two prompts (i.e. ‘What was the most recent DISTANCE displayed?’ and ‘What was the most recent ANIMAL displayed?’) along with a list of options to choose from. The correct response is the word colored in red (i.e. ‘YARD’ and ‘HORSE’).


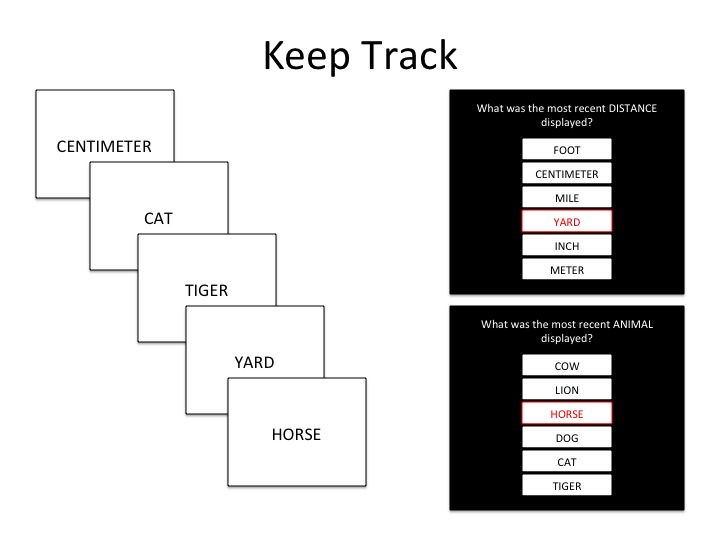


*Episodic memory.* Paired associates, immediate and delayed, represent the two episodic memory tests. In this task, a list of six unrelated word pairs are presented. At recall, only the first word from each pair is presented. Participants must type the second word in the pair. This procedure is repeated with a second list of six-word pairs. Approximately 20-30 minutes later, the first word from all twelve-word pairs is presented. Participants must type the second word in the pair.

*Fluid intelligence.* Fluid intelligence was measured using two tests: letter and number series. Letter series requires the identification of a pattern in a set of letters and complete 15 problems in 7 minutes, with each problem increasing in difficulty^9^. In the example figure below, the participant would need to identify the 4-letter series that follows a different pattern. The correct option is circled in red.


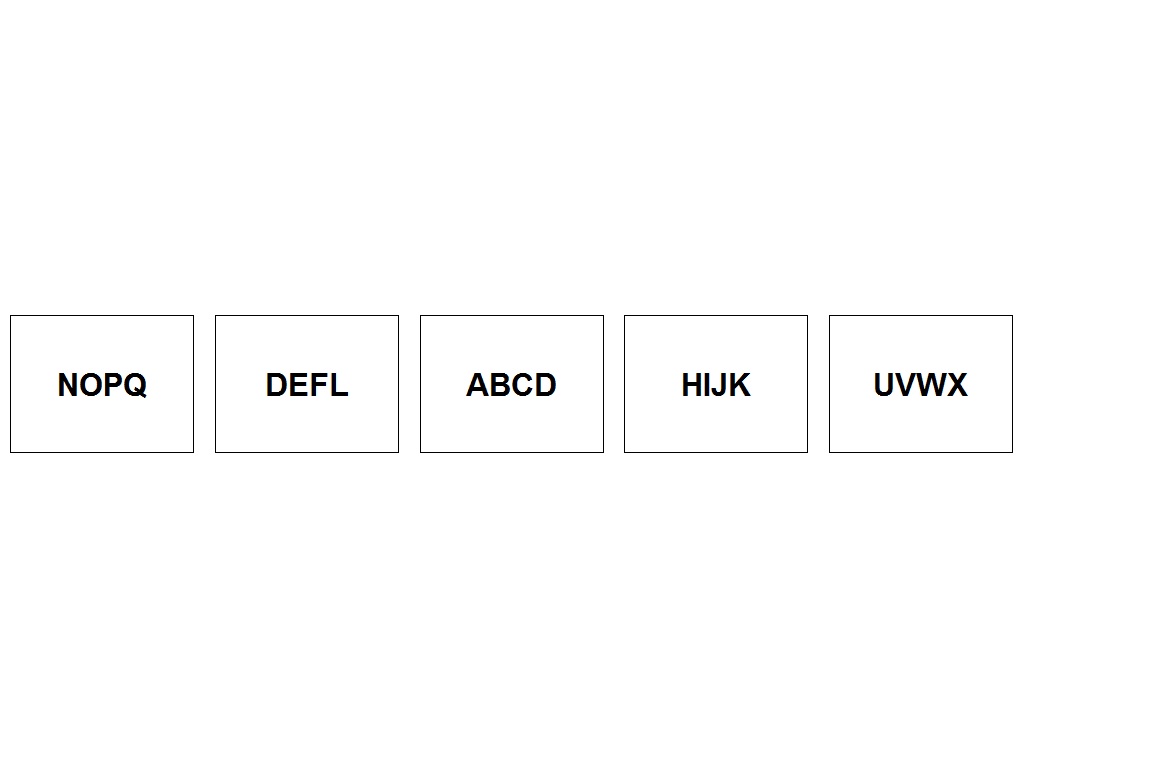


Number series presents a sequence of numbers and requires the identification of the next number in the sequence from a set of options; 10 problems are presented in 5 minutes^10^. In the example figure below, a participant sees the sequence of numbers and is then asked to select the correct response. The correct option is circled in red.


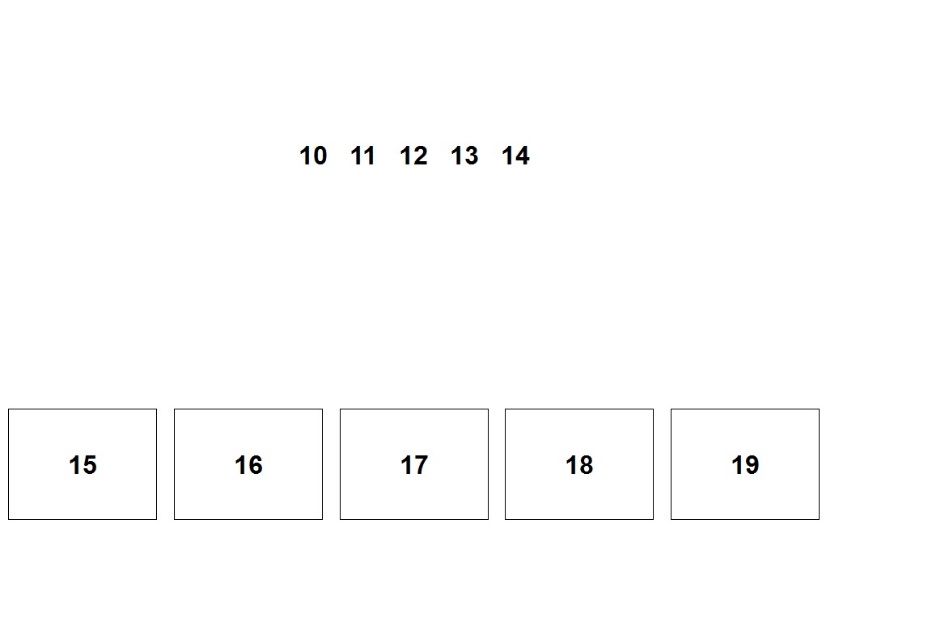


*Working memory.* Working memory was measured with rotation span and symmetry span. Shortened versions of both tests were administered, which have been verified to retain the [psychometric](https://www.sciencedirect.com/topics/medicine-and-dentistry/psychometry) properties of the longer versions^7^. Rotation span required the participant to remember a series of items while maintaining accuracy on another task^11^. Participants recalled a sequence of short and long arrows radiating from the center of the screen against a background letter-rotation task. The letter-rotation task presented a normal or mirror-reversed G, F, or R, rotated at 0°, 45°, 90°, 135°, 180°, 225°, 270°, or 315°. The task was to mentally rotate the letter, and then to indicate whether the letter was normal (True — approximately 50% of trials) or mirror reversed (False — approximately 50% of trials). Immediately after a response, the participant pressed a key clearing the screen for 0.5 s and was presented a short or long arrow rotated at 0°, 45°, 90°, 135°, 180°, 225°, 270°, or 315°. After 1 s, the arrow disappeared and another letter or the recall cue appeared instructing the participant to recall all the arrows from the preceding displays in the order they appeared. Participants were told to maintain accuracy on the letter-rotation task at 85% or higher. The time limit for the rotated letter judgment was individualized and determined by practice phase performance. Set sizes ranged from two to nine letter/arrow displays per trial (8 trials total). The figure below presents an example of a rotation span trial.


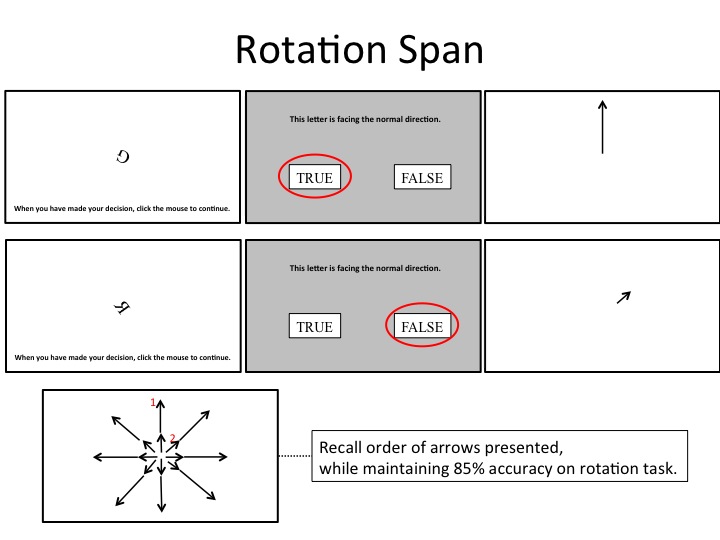


Symmetry span required a participant to remember a series of items while maintaining accuracy on another task^12^. On each trial a 4 × 4 grid was presented in which one of the 16 possible locations was filled in red (650 ms each for 3–6 locations). Participants were asked to remember the location of the red squares. Between each location presentation, participants were shown an 8 × 8 grid of black and white rectangles. They were asked to determine whether the grid was symmetric about the vertical axis (i.e., left half matches right). After all spatial locations were presented, participants were asked to reproduce the spatial locations in the order in which they were presented. Participants are told to maintain accuracy of identifying symmetry at 85% or higher and were shown their accuracy at the end of each trial. The time limit for the symmetry judgment was individualized and determined by practice phase performance. There was a total of 8 trials. The figure below presents an example of a symmetry span trial.


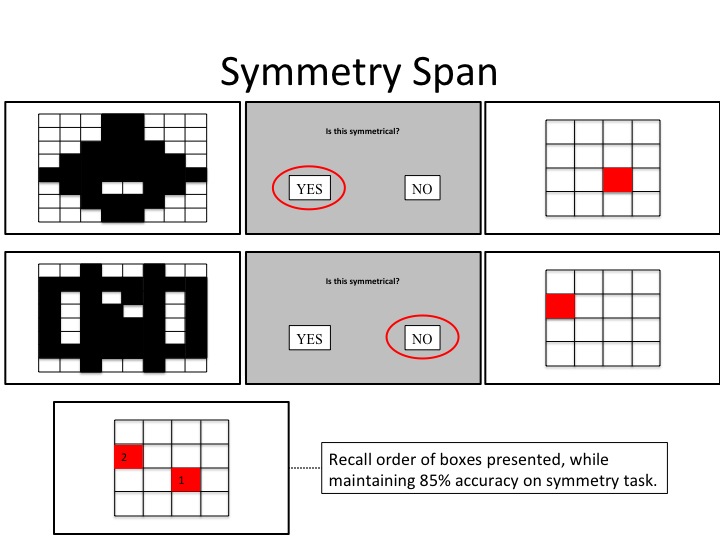


*Executive function.* Executive function was measured by the Stroop task, which is a measure of inhibition^13^. In this task, subjects are asked to indicate the color of each word stimulus. The stimulus may be congruent, that is the word meaning and text color match, or incongruent, if the word meaning and text color differ. The figure below shows an example trial from the Stroop task.


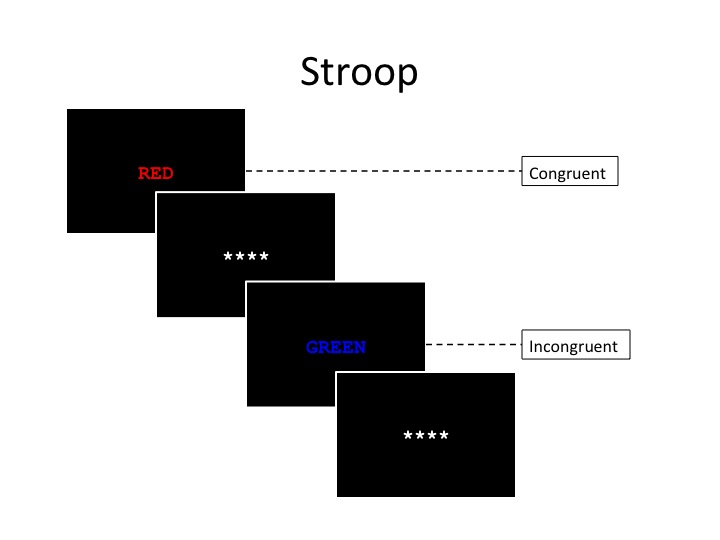


**Supplementary Note 3. Verifying random assignment at pre-intervention.**

The percent of males and females in each intervention group was equivalent ($\chi^{2}=0.11, p=0.74$). There were no age differences between the groups (*t* = 0.14, *p* = 0.89). Across the three educational levels listed in Table 1, there were no differences between the two intervention groups ($\chi^{2}=0.92, p=0.63$). And a test for the number of participants in each Air Force rank did not differ by intervention group ($\chi^{2}=7.57, p=0.37$). These results provide a first check for inferring that observed group differences are due to the intervention and not pre-existing characteristics of the groups.

A second check to verify random assignment examines group differences for the physical fitness and cognitive batteries administered at pre-intervention. If pre-intervention performance measures differ by group, and post-intervention differences between groups are observed, it is challenging to determine if those post-intervention differences are carryover effects from pre-intervention differences, due to the efficacy of the intervention alone or due to a combination of carryover and intervention effects. The ANOVA model, with the pre-intervention measure as the outcome and intervention group as the predictor, tests for group differences. The pre-intervention means for all 26 fitness measures (Supplementary Table 2) do not differ between the exercise plus supplement and exercise plus placebo group (all *p*-values > 0.05; all Cohen’s *d* < 0.25; all *Bf* < 1.0). The pre-intervention means for 15 of the 16 cognitive measures (Supplementary Table 3) do not differ between the exercise plus supplement and exercise plus placebo group (all *p*-values > 0.05; all Cohen’s *d* < 0.25; all *Bf* < 1.0). For Paired Associates Immediate Recall, the exercise plus placebo group has a higher mean than the exercise plus supplement group (*p*-value = 0.02; Cohen’s *d* = 0.39; *Bf* = 2.23).

Overall, given the demographic similarity between intervention groups, and similar scores on 41 out of 42 measures of fitness and cognition at pre-intervention, there is strong evidence that assignment of participants into intervention groups was random. Therefore, observed group differences at post-intervention can be attributed to the intervention.

**Supplementary Note 4. Principal Components Analysis.**

Supplementary Table 4 includes the factor scores for each physical fitness variable and the 5 components derived from PCA. The power component accounts for 27% of the variance and is comprised of abdominal circumference, lean muscle mass, sled rope pull, sled push and pull, rotation smash ball, weight and Wingate upper body. Strength and endurance, which accounts for 25% of the variance, includes percentage of body fat, the Modified Illinois Agility test, pull ups, push-ups, sit ups, standing long jump, VO_2_ max and Wingate lower body. Mobility and stability accounts for 12% of the variance and is indexed by the lateral and supine bridge and the lower and upper Y balance test. Blood pressure (BP) captures 7% of the variance and is composed of diastolic and systolic blood pressure. Finally, heart rate (HR) comprises 6% of the variance and is determined by maximum and resting heart rate.

Supplementary Table 5 includes the factor scores for each cognitive variable and the 5 components derived from PCA. Short term memory (STM), which accounts for 16% of the variance, includes Immediate Free Recall Words and Pictures and Keep Track of Words. Episodic memory (EM) accounts for 15% of the variance and includes immediate and delayed recall of Paired Associates. Fluid intelligence (Gf) accounts for 14% of the variance and includes Number Series and Letter Sets. Working memory (WM) accounts for 14% of the variance and includes Rotation and Symmetry Span. Executive function (EF) accounts for 13% of the variance and includes Stroop congruent and incongruent responses. Reaction time measures and processing efficiency were not included in the PCA but were included as part of the cognitive battery.

**Supplementary Note 5. Selectivity of interventions.**

The Bayes factors results that demonstrated the efficacy of the interventions in improving the fitness and cognitive measures are further leveraged to demonstrate selective improvements within and between interventions. Supplementary Table 9 lists all Bayes factors for both interventions for the physical fitness and cognitive measures. Two conclusions can be drawn from this table.

First, according to the standard Bayes factor criteria, the exercise plus placebo intervention decisively (*Bf* > 150) improved strength and endurance, mobility and stability and fluid intelligence accuracy; strongly (20 ≤ *Bf* < 150) improved power; positively (3 ≤ *Bf* < 20) improved paired associates accuracy and processing efficiency; slightly (1 ≤ *Bf* < 3) improved heart rate; and did not (*Bf* < 1) improve blood pressure, episodic memory, fluid intelligence reaction time or working memory. The exercise plus supplement intervention decisively improved strength and endurance, mobility and stability, heart rate, power, fluid intelligence accuracy, executive function reaction time and processing efficiency; positively improved blood pressure and paired associates; slightly improved fluid intelligence reaction time and working memory; and did not improve episodic memory or executive function accuracy.

Second, the two ‘Ratio’ columns in Table 8 demonstrate the intervention with a larger effect on the physical fitness and cognitive measures. The exercise plus placebo intervention, relative to the exercise plus supplement, was more effective at improving strength and endurance, fluid intelligence accuracy and episodic memory. The exercise plus supplement intervention, relative to the exercise plus placebo intervention, improved 8 physical fitness and cognitive domains: heart rate, power, blood pressure mobility and stability, executive function reaction time, processing efficiency, working memory and fluid intelligence reaction time. For the remaining two measures in Table 8, short term memory and executive function accuracy, the ratio of Bayes factors is not worth interpreting because neither intervention improved the outcomes (i.e. the individual Bayes factors are both less than 1.0).

**Supplementary Note 6. Passive control from different study.**

For the ANCOVA, a set of 74 control participants from another study designed to assess gains in cognition due to interventions was included to provide a true baseline reference group. The control sample was matched to the participants of the current study on important variables at pre-intervention, including height, weight, sex, maximum heart rate and VO_2_ max. These matched controls are the ‘Control’ group in Table 2. The matched controls only completed the pre- and post-cognitive assessment. In the 16-week interval between testing, participants did not engage in fitness training, consume a nutritional beverage or otherwise complete an intervention. The cognitive scores at pre-intervention did not differ among the passive control, exercise plus placebo and exercise plus supplement intervention (all *p*-values > 0.05; all Cohen’s *d* < 0.25; all *Bf* < 1.0).

There were differences among the passive control, exercise training plus placebo and exercise training plus nutritional supplement for fluid intelligence accuracy (*F* = 6.2, *df* = (2,189), *p* = 0.0026, *Bf* = 9.43), working memory (*F* = 4.1, *df* = (2,191), *p* = 0.018, *Bf* = 1.86) and executive function reaction time (*F* = 6.8, *df* = (2,190), *p* = 0.0015, *Bf* = 16.5). Follow up analyses demonstrate both the exercise training plus placebo and the exercise training plus nutritional supplement each improve relative to the passive control for fluid intelligence accuracy and executive function reaction time; but there is no difference between the exercise training plus placebo and exercise training plus nutritional supplement. For working memory, the exercise training plus nutritional supplement differs from both the passive control and the exercise training plus placebo; but there is not a difference between the exercise training plus placebo and the passive control. Running the ANCOVA model without the passive control demonstrates the exercise training plus nutritional supplement improves fluid intelligence reaction time (*F* = 2.28, *df* = (1,139), *p* = 0.13, Cohen’s *d* = 0.25, *Bf* = 1.08) and working memory (*F* = 6.81, *df* = (1,142), *p* = 0.01, Cohen’s *d* = 0.43, *Bf* = 4.16).

There were no effects for episodic memory (*F* = 0.96, *df* = (2,186), *p* = 0.38, *Bf* = 0.13), paired associates (*F* = 1.66, *df* = (2,164), *p* = 0.19, *Bf* = 0.25), executive function accuracy (*F* = 0.97, *df* = (2,190), *p* = 0.38, *Bf* = 0.18) or processing efficiency (*F* = 3.1, *df* = (1,141), *p* = 0.08, Cohen’s *d* = 0.29, *Bf* = 0.57).

**Supplementary References**

1 Bergeron, M. F. *et al.* Consortium for Health and Military Performance and American College of Sports Medicine consensus paper on extreme conditioning programs in military personnel. *Current sports medicine reports* **10**, 383-389 (2011).

2 Friel, J. *Total heart training: customize and maximize your workout using a heart rate monitor*. (Ulysses Press, 2009).

3 Kaufman, K. R., Brodine, S. & Shaffer, R. Military training-related injuries: surveillance, research, and prevention. *American Journal of Preventative Medicine* **18**, 54-63 (2000).

4 Nye, N. S., Pawlak, M. T., Webber, B. J., Tchandia, J. N. & Milner, M. R. Description and rate of musculoskeletal injuries in Air Force basic military trainees, 2012-2014. *Journal of Athletic Training* **58**, 858-865 (2016).

5 Unsworth, N., Spillers, G. J. & Brewer, G. A. The contributions of primary and secondary memory to working memory capacity: an individual differences analysis of immediate free recall. *Journal Experimental Psychology: Learning, Memory and Cognition* **36**, 240-247 (2010).

6 Salthouse, T. A. Speed mediation of adult age differences in cognition. *Developmental Psychology* **29**, 722 (1993).

7 Foster, J. L. *et al.* Do the effects of working memory training depend on baseline ability level? *Journal of Experimental Psychology: Learning, Memory and Cognition* **43**, 1677-1689 (2017).

8 Yntema, D. B. Keeping track of several things at once. *Hum Factors* **5**, 7-17 (1963).

9 Ekstrom, R. B., French, J. W., Harman, H. H. & Dermen, D. Manual for kit of factor-referenced cognitive tests. 109-113 (Educational Testing Service, Princeton, 1976).

10 Bernreuter, R. G. & Goodman, C. H. A study of the Thurstone Primary Mental Abilities Tests applied to freshman engineering students. *Journal of Educational Psychology* **32**, 55-60 (1941).

11 Shah, P. & Miyake, A. The separability of working memory resources for spatial thinking and language processing: an individual differences approach. *Journal of Experimental Psychology: General* **125**, 4-27 (1996).

12 Unsworth, N., Redick, T. S., Heitz, R. P., Broadway, J. M. & Engle, R. W. Complex working memory span tasks and higher-order cognition: a latent-variable analysis of the relationship between processing and storage. *Memory* **17**, 635-654 (2009).

13 Stroop, J. R. Studies of interference in serial verbal reactions. *Journal of Experimental Psychology* **18**, 643-662 (1935).
